# Consistency and variability of cocrystals containing positional isomers: the self-assembly evolution mechanism of supramolecular synthons of cresol–piperazine

**DOI:** 10.1107/S2052252519012363

**Published:** 2019-10-09

**Authors:** Na Wang, Xin Huang, Lihang Chen, Jinyue Yang, Xin Li, Jiayuan Ma, Ying Bao, Fei Li, Qiuxiang Yin, Hongxun Hao

**Affiliations:** a National Engineering Research Center of Industrial Crystallization Technology, School of Chemical Engineering and Technology, Tianjin University, Tianjin 300072, People’s Republic of China; b Collaborative Innovation Center of Chemical Science and Engineering, Tianjin 300072, People’s Republic of China; c Key Laboratory for Green Chemical Technology of the Ministry of Education, R&D Center for Petrochemical Technology, Tianjin University, Tianjin 300072, People’s Republic of China

**Keywords:** cocrystal consistency, cocrystal variability, supramolecular synthons, theoretical calculations, quantum chemistry, intermolecular interactions, co-crystals, hydrogen bonding, density functional theory, lattice energy

## Abstract

The disposition of functional groups can induce variations in the nature and type of interactions and hence affect the molecular recognition and self-assembly mechanism in cocrystals. To better understand the formation of cocrystals on a molecular level, the effects of disposition of functional groups on the formation of cocrystals were systematically and comprehensively investigated using cresol isomers (*o*-, *m*-, *p*-cresol) as model compounds. Consistency and variability in these cocrystals containing positional isomers were found and analyzed. The structures, molecular recognition and self-assembly mechanism of supramolecular synthons in solution and in their corresponding cocrystals were verified by a combined experimental and theoretical calculation approach. It was found that the heterosynthons (heterotrimer or heterodimer) combined with O—H⋯N hydrogen bonding played a significant role. Hirshfeld surface analysis and computed interaction energy values were used to determine the hierarchical ordering of the weak interactions. The quantitative analyses of charge transfers and molecular electrostatic potential were also applied to reveal and verify the reasons for consistency and variability. Finally, the molecular recognition, self-assembly and evolution process of the supramolecular synthons in solution were investigated. The results confirm that the supramolecular synthon structures formed initially in solution would be carried over to the final cocrystals, and the supramolecular synthon structures are the precursors of cocrystals and the information memory of the cocrystallization process, which is evidence for classical nucleation theory.

## Abstract 2

The consistency and variability as well as self-assembly evolution mechanism of cresol–piperazine cocrystals are investigated by experiments and molecular simulation.

## 1. Introduction   

Supramolecular chemistry is a very active area (Zhao & Truhlar, 2007 ▸). An important part of crystal engineering and supramolecular chemistry (Wouters & Quéré, 2012 ▸) – cocrystals – which are single-crystal structures composed of two or more components in a certain stoichiometric ratio with no proton transfer between components and are formed by noncovalent bonds, have been known for a long time (Wang *et al.*, 2017 ▸, 2018*a* ▸,*b* ▸). Inspired by supramolecular self-assemblies in nature (Matsumoto *et al.*, 2018 ▸), organic cocrystals have been applied to many fields such as engineering pharmaceutical solids (Childs *et al.*, 2004 ▸; Almarsson & Zaworotko, 2004 ▸) and organic cocrystal materials (Zhu *et al.*, 2015 ▸; Sun *et al.*, 2018 ▸) with the help of noncovalent bonding.

Noncovalent interactions are relatively weak and flexible compared with other bonds in molecular constructions, *e.g.* covalent bonds, which makes them useful in crystal engineering (Varughese *et al.*, 2015 ▸). Cocrystallization of different chemical components in the same crystal structure is an important phenomenon in science and technology (Zhang *et al.*, 2013*a* ▸,*b* ▸). The complexity of organic molecules – irregular shapes, chirality, flexibility and hydrogen bonding – makes their cocrystallization fundamentally different from that of metals and other inorganic substances (Zhang *et al.*, 2013*a* ▸,*b* ▸). In order to further understand and explore the formation mechanism of cocrystals (or complexes) of organic molecules, especially isomers, many researchers have done extensive research on the cocrystals of different organic molecules (Varughese *et al.*, 2015 ▸; Saha & Desiraju, 2018 ▸; Portalone & Rissanen, 2018 ▸; Sánchez-Guadarrama *et al.*, 2016 ▸; Wang *et al.*, 2018*a* ▸,*b* ▸, 2019 ▸). However, most of these studies focused on either describing the crystal structure of the molecular complex obtained in detail, or studying the properties of the cocrystal from the crystal structure and/or the type of synthons by using spectroscopic analysis. In addition, the evolution of the solute molecules in solution during the formation of cocrystals has not been well studied. The reasons for the same and/or different properties of cocrystals formed by the same coformer (which have the same functional groups) with positional isomerism have not been systematically studied. Although the Δp*K*_a_ rule has been proposed to explain or evaluate the formation of cocrystals (Musumeci *et al.*, 2011 ▸; Childs *et al.*, 2007 ▸; Johnson & Rumon, 1965 ▸; Hathwar *et al.*, 2010 ▸), it cannot be used to explain many phenomena in the cocrystallization process. In reality, different isomers of the same molecule often exhibit different results, though they also exhibit some similarities such as physical properties, synthon patterns *etc*.

The three pillars for advancement in modern crystal engineering, crystallography, spectroscopy and computation are widely applied in this field (Saha & Desiraju, 2018 ▸). Spectral data are usually related to the chemical features and a given supramolecular synthon is associated with its corresponding signals (Parveen *et al.*, 2005 ▸; Du *et al.*, 2015 ▸; Davey *et al.*, 2006 ▸). As a supplementary verification method for experimental work, computational simulation can complete some aspects that are difficult to achieve experimentally. In addition, as the smallest unit of the crystal structure, the synthon provides important information on the process of the crystallization (Desiraju, 2002 ▸), and it can be directly detected by spectroscopy (Parveen *et al.*, 2005 ▸; Davey *et al.*, 2006 ▸; Mukherjee *et al.*, 2014 ▸) and indirectly verified by theoretical computation (Price, 2009 ▸).

In this work, to understand the formation of cocrystals on a molecular level and to help the design and development of cocrystal materials, the effects of disposition of functional groups on the formation of cocrystals were investigated by using cresol isomers (*o*-, *m*-, *p*-cresol, herein abbreviated to OC, MC and PC, respectively) and piperazine (PP) as model compounds (see Fig. S1 of the supporting information). The consistency and variability exhibited by cocrystals formed by cresol isomers with the same coformer were summarized and analyzed. Firstly, the crystal structures of the cocrystals obtained are studied in detail, and the intermolecular interactions present are analyzed and compared by Hirshfeld surface (HS) analysis. Secondly, the structures of the supramolecular synthons obtained from the cocrystal structures were analyzed by liquid and solid IR as well as ^1^H NMR spectroscopy; the structures of the supramolecular synthons were verified by theoretical computation. Thirdly, with the help of molecular simulation, DFT calculations were performed to further investigate consistency and variability. The energy evaluation and the quantitative analysis of charge transfers and molecular electrostatic potential surfaces (MESP) were also applied to elucidate consistency and variability. Finally, the evolution pathway of the synthons in solution during the formation of cocrystals were investigated using Process Analysis Tools/technologies (PAT), and classical nucleation theory was supported by the data obtained.

## 2. Results and discussion   

### 2.1. Crystal structures and structural consistency and variability   

#### 2.1.1. Crystal structures and molecular arrangements   

**Figure 1:**
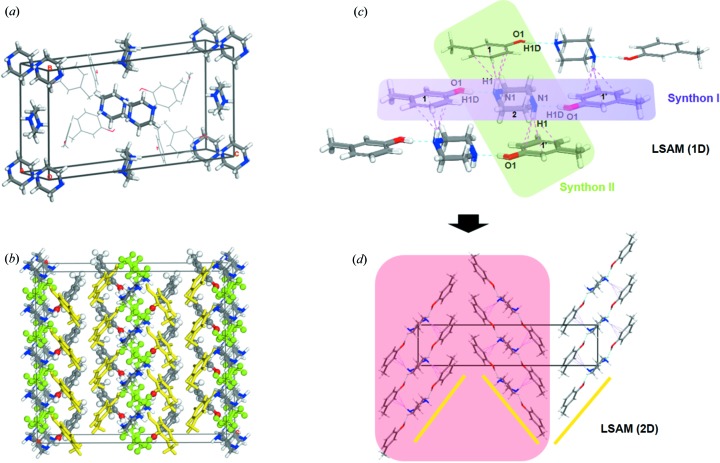
The crystal structure, packing model and intermolecular interactions of MC_PP. (*a*) Unit cell of MC_PP. (*b*) 3D supramolecular packing model in the supercell with 4 × 1 × 1. (*c*) LSAM (1D) constructed by amalgamation of Synthon I (supramolecular synthon highlighted in purple interacting via O—H⋯N hydrogen bonds) and Synthon II (supramolecular synthon highlighted in green interacting via N—H⋯π hydrogen bonds). (*d*) Two-dimensional LSAM structure (highlighted in red) constructed by an arrangement of the LSAMs (1D) along the *oac* plane. Purple dotted lines and blue dotted lines represent π⋯H and O—H⋯N hydrogen bonding, respectively.

Crystal data, data collection and structure refinement details are summarized in Table S2 of the supporting information. The crystal structure, packing model and intermolecular interactions of these three cocrystals are shown in Fig. 1 ▸ and Figs. S3 and S4 of the supporting information. A stable crystal structure is primarily guided by the principle of close-packing, according to which the protrusions of one molecule fit into the voids of another (Chakraborty *et al.*, 2018 ▸; Fábián & Kálmán, 1999 ▸; Kálmán *et al.*, 1993 ▸). From the crystal structures we can see that, in the unit cell, the PP molecules essentially constitute the edge portion of the entire unit cell and substantially determine the size and volume of the unit cell, while the cresol molecules fill the remaining spaces. In addition, the *m*-cresol_piperazine cocrystal (MC_PP cocrystal) and the *o*-cresol_piperazine cocrystal (OC_PP cocrystal) are formed in a 1:2 molar ratio of PP and MC molecules and/or two OC molecules, respectively. However, the asymmetric unit of the *p*-cresol_piperazine cocrystal (PC_PP cocrystal) contains one PC molecule and one PP molecule in a stoichiometric molar ratio of 1:1. More detailed structural information about the MC_PP, OC_PP and PC_PP cocrystals is provided in the supporting information.

In summary, the cocrystals of the cresol isomers are mainly assembled by hydrogen bonding (Table 1 ▸); this is consistent for all three cocrystals. Furthermore, from the analysis of the crystal structure, although MC_PP and OC_PP belong to different crystal systems, the two cocrystals are very similar in crystal structure, synthon pattern and long-range synthon Aufbau modules (LSAMs) (Ganguly & Desiraju, 2010 ▸; Mukherjee *et al.*, 2014 ▸; Dubey *et al.*, 2016 ▸), also demonstrating consistency. However, although PC has the same functional groups as MC and OC, the PC_PP cocrystal exhibits significant variability in structure, synthon pattern and LSAMs when compared with MC_PP and OC_PP. For MC_PP and OC_PP, there is one PP molecule and two MC or OC molecules in the asymmetric unit, whereas the asymmetric unit of PC_PP consists of only one PP molecule and one PC molecule. In addition, in the unit cells of MC_PP and OC_PP, the PP molecules occupy all the vertices of the unit cells and the MC or OC molecules fill the voids. Because the void size is not necessarily the same as the volume of cresol, they cannot be closely packed. The similar packing modes lead to comparable lattice energies of the two cocrystals (lattice energies are given in Table 2 ▸), 39.65 kcal mol^−1^ for MC_PP and 40.91 kcal mol^−1^ for OC_PP. Their melting points are also very close as shown in Fig. S11, 61.7°C for MC_PP and 60.7°C for OC_PP. However, in the unit cell of PC_PP, no molecule occupies any vertex position. The PP and PC molecules are closely packed in a way which leads to a much higher lattice energy of 83.41 kcal mol^−1^ for PC_PP than those of MC_PP and OC_PP, and the melting point of PC_PP (92.6°C) is also much higher than those of MC_PP and OC_PP. Moreover, there are two heterosythons with a stoichiometric ratio of 1:2 (one PP molecule to two MC or OC molecules) in both MC_PP and OC_PP. The two types of ternary synthons interact by O—H⋯N and N—H⋯π hydrogen bonds. However, for PC_PP, three different types of synthons are formed: two of which are heterosynthons with a stoichiometric ratio of 1:1 (one PP molecule to one PC molecule, interacting via O—H⋯N and N—H⋯π hydrogen bonds, respectively), whereas the third is a homosynthon interacting through N—H⋯N hydrogen bonds. These different types of supramolecular synthons interact by hydrogen bonding, leading to different 1D/2D LSAMs, as shown in Figs. 1 ▸(*c*) and 1(*d*), Figs. S3(*c*) and S3(*d*), and Figs. S4(*c*) and S4(*d*).

### 2.2. Possible self-assembly patterns of supramolecular synthons in solution   

**Figure 2:**
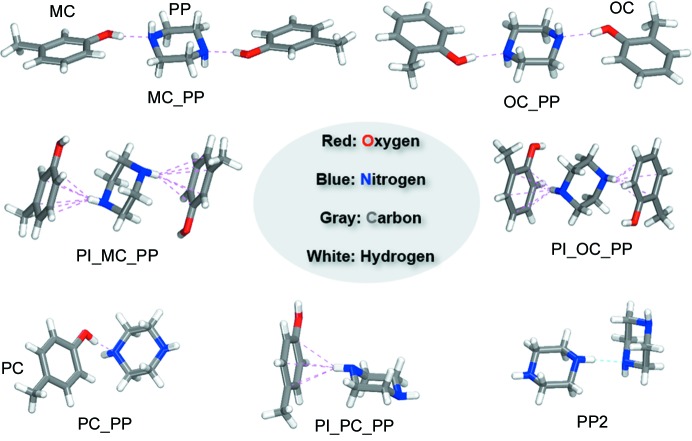
Supramolecular synthons used for the recognition in the cresol isomer cocrystals.

Supramolecular synthon patterns and their strength are significant factors in stabilizing molecular units in crystal structures, and hence it is worth analysing their contributions toward structure formation (Varughese *et al.*, 2015 ▸). Synthon structures in solution can be imaged directly by spectroscopic studies (Parveen *et al.*, 2005 ▸; Mukherjee *et al.*, 2014 ▸) and indirectly through computational methods (Price, 2009 ▸; Thakur *et al.*, 2015 ▸) or by analysing experimental crystal structures (Sreekanth *et al.*, 2007 ▸; Mukherjee & Desiraju, 2014 ▸). IR and NMR spectroscopy are often used to analyze synthon structures (Parveen *et al.*, 2005 ▸; Mukherjee *et al.*, 2014 ▸). In general, synthons are kinetic units (Desiraju, 2002 ▸) and can be affected by concentration of solvent, temperature and pressure. According to classical nucleation theory, the synthons are likely to form initially in solution, and then carry over into the final product. In view of the patterns of supramolecular synthons in the cocrystal structures, seven possible types of self-assembly patterns of synthons (Fig. 2 ▸) in solution and in cocrystal are discussed in the context of detection, calculation and verification: two modes through two different hydrogen bonds [O(1)—H(1D)⋯N(1) and N(1)—H(1)⋯π] for MC_PP, another two modes through two different hydrogen bonds [O(1)—H(1 A)⋯N(1) and N(1)—H(1)⋯π] for OC_PP, and three different modes through hydrogen bonds [O(1)—H(1D)⋯N(1) and N(2)—H(2)⋯π of the hetersynthon and N(1)—H(1)⋯N(2) of the homosynthon] for PC_PP. First of all, to find the most probable and dominant supramolecular synthon patterns, changes in the molecular functional group vibrations in the cocrystal and in solution were investigated to understand the chemical basis for dimers and/or trimers with the help of spectroscopic studies (IR, Raman and NMR spectroscopy) and computational methods. Meanwhile, the dissociation energies of various possible synthons in cocrystals and in solution were computed to find the thermodynamically stable patterns. Finally, since the monomer of the host and guest molecules as well as the dimers and/or trimers in the solution will reach thermodynamic equilibrium under certain temperature and concentration conditions, the formation processes of synthons and cocrystals were also detected by PAT to investigate the evolution pathway of the synthons.

#### 2.2.1. IR features of various synthons   

**Figure 3:**
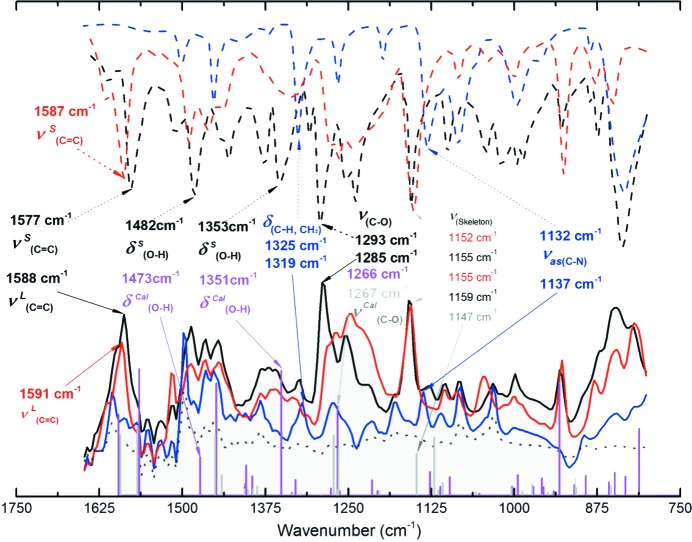
Characteristic absorption peaks corresponding to solid FTIR data (dotted black curve for MC_PP, red for MC and blue for PP), liquid ATR-FTIR data (solid black curve for MC_PP trimer, red for MC, blue for PP in toluene, and gray dotted curve for pure toluene) and the computational results in toluene (light purple vertical line for t(MCPP) in toluene solution and light gray vertical line for the t(PI_MCPP) in toluene solution). Symbols: ν: stretching, δ: in-plane bending; (superscripts) *L*: ATR-FTIR data; *S*: FTIR data; *cal*: computational data; (subscripts) *as*: antisymmetric.

To investigate the chemical nature of the supramolecular synthons in solution and solid state through IR spectroscopy, the changes of the molecular functional group vibrations in the cocrystals were compared with the single-component systems. The IR results by spectroscopic studies and computational methods are shown in Figs. 3 ▸ and S6, and the Raman spectroscopic results are shown in Fig. S7. The MC_PP cocrystal was taken as an example, more detailed information is provided in the supporting information. Comparing the IR spectra of the two supramolecular synthons calculated in gas, we can see that all characteristic peaks on the black line (experimental results) can be found in the calculated spectra (light purple line plus light blue line) in Fig. S6a. In particular, the light purple curve is more similar to the experimental data than the light blue curve. This indicates that there are two weak interaction modes [MC_PP with O(1)—H(1D)⋯N(1) and PI_MC_PP with N(1)—H(1)⋯π] in the solid state MC_PP cocrystal, which is consistent with the results of single-crystal structure analysis. It also confirms that the results of the calculation are reliable. Moreover, the heterotrimer synthons combined with the O(1)—H(1D)⋯N(1) hydrogen bonds play a dominant role in the MC_PP cocrystals. Fig. 3 ▸ displays the vibration modes of the IR fingerprint region. The solid and liquid experimental IR spectra and the computed IR results in toluene solution are compared in this figure. This further supports the existence of a heterotrimer of MC and PP molecules in toluene solution, mainly in the form of t(MCPP) assembled via O—H⋯N hydrogen bonds. t(MCPP) and/or MC_PP are the dominant synthons in toluene solution and the solid state; more detailed information can be found in supporting information.

Fig. S6 shows the solid FTIR spectra, ATR-FTIR spectra in toluene and the computed spectra of MC_PP, OC_PP, PC_PP and their components, respectively. Unsurprisingly, similar results can be obtained for OC_PP and PC_PP. For OC_PP, there are two weak interaction modes [OC_PP synthon with O(1)—H(1 A)⋯N(1) and PI_OC_PP synthon with N(1)—H(1)⋯π], which are consistent with the results of single-crystal structure analysis. Additionally, the heterotrimer synthons [t(OCPP)] with the O(1)—H(1 A)⋯N(1) mode play a dominant role in the OC_PP cocrystals, as well as in toluene solution. Nevertheless, for PC_PP cocrystals, there are three interaction modes [PC_PP heterosynthon combined with O(1)—H(1D)⋯N(1), PI_PC_PP heterosynthon combined with N(2)—H(2)⋯π and PP2 homosynthon combined with N(1)-H(1)⋯N(2)], which are consistent with the results of single-crystal structure analysis. Moreover, the PC_PP heterosynthon combined with O(1)—H(1D)⋯N(1) and PP2 homosynthon combined with N(1)—H(1)⋯N(2) are the primary weak interaction modes. However, the homodimer [d(PP2)] is not the dominant synthon in toluene because of its weaker intermolecular interaction strength and lower quantity than the heterodimer [d(PCPP) with O(1)—H(1D)⋯N(1)]. As a consequence, we can infer that trimers and/or dimers are initally formed in toluene solution before the nucleation and growth of cocrystals and are then carried over into the final products, as classical nucleation theory assumes. The heterotrimers and/or heterodimers combined with O—H⋯N are the most critical and dominant synthons in toluene solution. Also, the probability of N—H⋯π hydrogen bonding synthons in solution is very low and it is very likely that the π⋯H hydrogen bonding enhances the stability of the solid during cocrystal formation. This further supports the continuation of the molecular state in solution into the solid state, which is in agreement with classical nucleation theory.

#### 2.2.2. ^1^H NMR features of various synthons   

**Figure 4:**
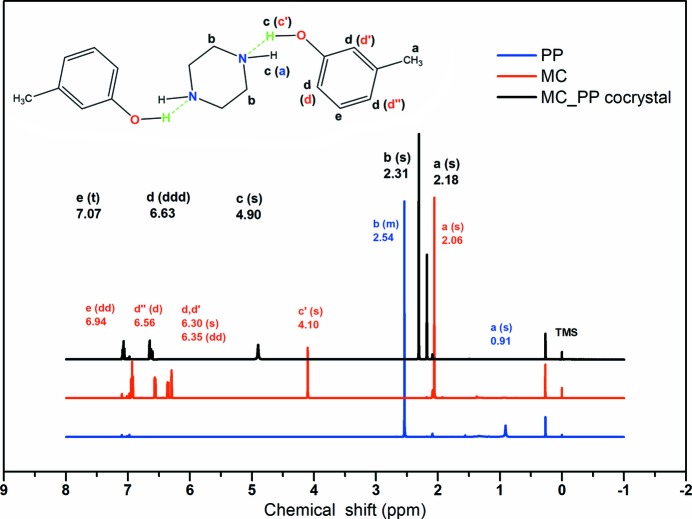
^1^H NMR spectra of MC_PP, MC and PP in toluene-*d*_8_. ^1^H NMR (MC_PP, 500 MHz, toluene-*d*_8_, 25°C, TMS): δ = 7.07 (t, 2 H; = CH-), 6.63 (ddd, 6 H; = CH-), 4.90 (s, 4 H; OH+NH), 2.31 (s, 8 H; CH_2_), 2.18 ppm (s, 6 H; CH_3_). ^1^H NMR (MC, 500 MHz, toluene-*d*_8_, 25°C, TMS): δ = 6.94 (dd, 1 H; = CH-), 6.56 (d, 1 H; = CH-), 6.35 (dd, 1 H; = CH-), 6.30 (s, 1 H; = CH-), 4.10 (s, 1 H; OH), 2.06 ppm (s, 3 H; CH_3_). ^1^H NMR (PP, 500 MHz, toluene-*d*_8_, 25°C, TMS): δ = 2.54 (m, 4 H; = CH_2_-), 0.91 (s, 1 H; NH).

In view of the fact that almost all the above interactions involve hydrogen atoms, and in order to prove that cresol molecules and piperazine molecules in toluene solution are mainly in the form of heterotrimers or heterodimers combined with O—H⋯N, ^1^H NMR spectra of the cocrystals in toluene-*d*_8_ solution were collected. Typically, for ^1^H NMR, chemical shift values indicatea particular chemical environment of the protons, and the peak area, which is the height of the integral curve, is proportional to the number of protons in that particular chemical environment (Pan & Zhang, 2009 ▸). The results are shown and listed in Figs. 4 ▸ and S8 and Table S3, which show that the cresol molecules and PP molecules exist in different stoichiometric ratios of the multimer in toluene solution before the formation of cocrystals. For MC_PP and OC_PP, both are present mainly in the form of heterotrimers with O—H⋯N hydrogen bonding in toluene solution (two MC molecules and/or two OC molecules combined with one PP molecule). Whereas PC_PP exists mainly in the form of a heterodimer with O—H⋯N hydrogen bonding in toluene solution (one PC molecule and one PP molecule). A more detailed analysis of the ^1^H NMR features of various synthons of MC_PP, OC_PP and PC_PP is provided in the supporting information.

Therefore, we can confirm that a sufficient number of heterotrimers or heterodimers in the form of heterosynthons in the cocrystal structure are already formed in toluene before the formation of cocrystals during cooling crystallization. In other words, the heterotrimers and/or heterodimers in solution will carry over into the supramolecular synthons of the solid cocrystal. Meanwhile, the MC_PP and OC_PP cocrystals exhibit consistency in the formation of heterotrimers and/or heterosynthons with the same O—H⋯N hydrogen bonding interaction and the same stoichiometric ratio (one PP molecule to two MC or OC molecules). On the other hand, PC_PP exhibits variability of the supramolecular synthons with a 1:1 stoichiometric ratio and interacts via O—H⋯N hydrogen bonds.

#### 2.2.3. Intermolecular interaction energy of synthons   

It is well known that the lower the energy of a substance, the more stable its state under certain conditions. Hence, the supramolecular synthons in toluene solution, as well as the lattice energy of the cocrystals (Bisker-Leib & Doherty, 2001 ▸) were analyzed from an energy perspective. All the structures [including host molecules, guest molecules (coformers) and the complexes (supramolecular synthons)] mentioned in this context were initially taken from the refined single-crystal structures and were fully optimized to stable structures in the gas phase and in toluene solution on an affordable DFT level together with D3 dispersion correction. The interaction energy Δ*E* of the optimized system was calculated by the same level, with the BSSE correction in the supramolecular approach, using equation (1): 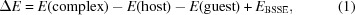
where the energy *E* is the total electronic energy.

Considering the crystal structures of these cocrystals and the spectral analysis results, 14 supramolecular synthons were investigated: 7 synthons in the gas phase and 7 synthons in toluene solution. The calculation results are shown in Table 2 ▸.

From Table 2 ▸, it can be seen that the computed results of the interaction energy are in good agreement with the conclusions obtained by the spectral and structure analyses. Whether in the gas phase or in toluene environment, the heterodimers or heterotrimers with O—H⋯N hydrogen bonds have the lowest energy and are the energy-dominant synthons. From an interaction energy point of view, the formation difficulty and stability of MC_PP and OC_PP are consistent. Nevertheless, the energies of the supramolecular synthons of PC_PP are obviously different from the corresponding supramolecular synthons of MC_PP and OC_PP, which shows variability from an energy perspective. A more detailed energy analysis can be found in the supporting information.

Therefore, the interaction energy results demonstrate that the dominant heterosynthons (binary and ternary heterosynthons) combined with the O—H⋯N hydrogen bonds are the most stable synthons in toluene solution and in the cocrystal, and the interaction energy results are consistent with those obtained from the spectral and structure analyses. The synthons of OC_PP and MC_PP exhibit consistency with respect to energy and structure/synthon type, whereas PC_PP shows variability. Moreover, this further supports that the form of heterotrimers and/or heterodimers in solution will carry over into the cocrystal state and the interaction energies of the supramolecular synthons in the cocrystal are lower than those of the heterotrimers and heterodimer in toluene.

#### 2.2.4. Verification of the evolution pathway of heterodimers and/or heterotrimers during cocrystal formation using PAT   

**Figure 5:**
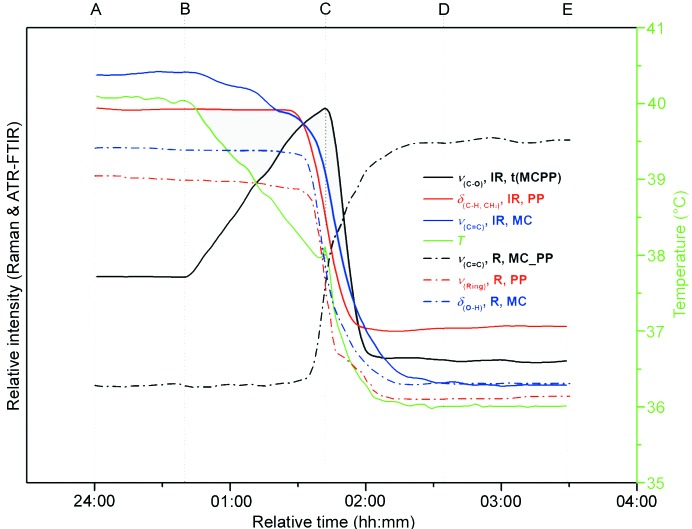
Changing trends of Raman and ATR-FTIR data during the cooling crystallization process of trimer verification experiments for MC_PP. R: Raman data; IR: ATR-FTIR data.

In this work, IR and Raman spectra were used to monitor the cocrystallization process *in situ*. Detailed information about IR and Raman spectra and the characteristic peaks of different compounds are given in the supporting information. Because of the influence of temperature and solute concentration, not all of the host molecules and coformers added to the system interact with each other, but a thermodynamic equilibrium will be reached in solution at a certain temperature and concentration. In order to understand the molecular recognition, self-assembly process and mechanism of these three cocrystal formation processes, PAT technology was used to monitor the cocrystal formation processes *in situ* under the above experimental conditions. The profiles of Raman data, ATR-FTIR data and temperature during the cocrystallization process are given in Figs. 5 ▸, S9 and S10. The verification of the evolution pathway of heterodimers and/or heterotrimers during cocrystal formation by PAT is described in the supporting information. From detection of the pathway, the formation process of cocrystals can be divided into three steps: (i) heterotrimer or heterodimer formation, (ii) cocrystal nucleation and (iii) cocrystal growth, as previously reported in the *m*-cresol_urea cocrystal system (Wang *et al.*, 2017 ▸).

Hence, this also shows that the structures of the heterotrimers or heterodimers that exist in solution will carry over into the corresponding cocrystals, which is consistent with classical nucleation theory.

### 2.3. Reasons for consistency and variability   

**Figure 6:**
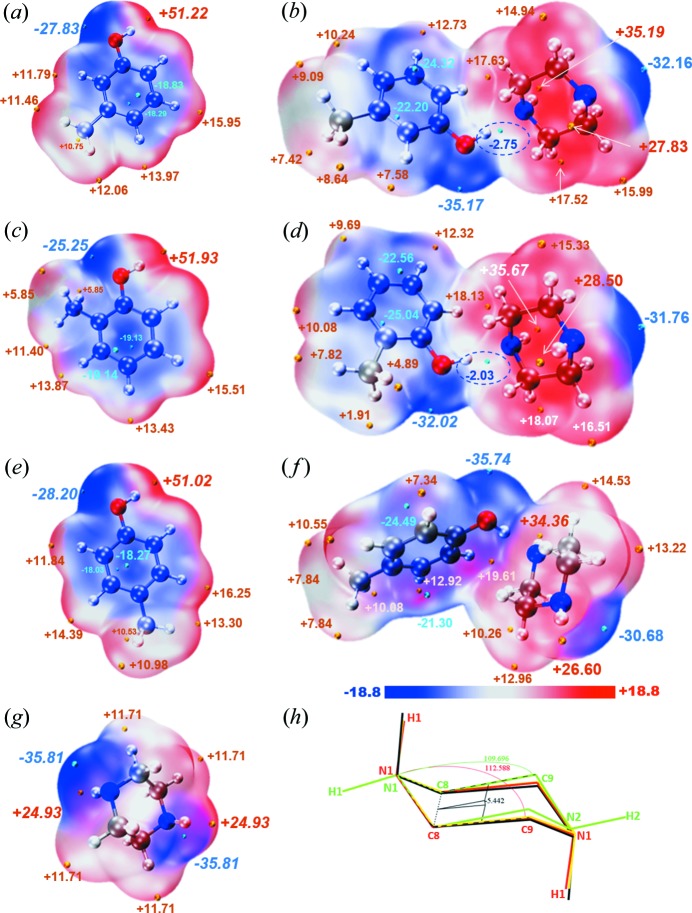
ESP-mapped molecular vdW surface of cresol isomers, a PP molecule and synthons with the ratio of 1:1. (*a*) MC ESP, (*b*) MC1PP ESP, (*c*) OC ESP, (*d*) OC1PP ESP, (*e*) PC ESP, (*f*) PC_PP ESP, (*g*) PP ESP and (*h*) deformation of PP molecules in three cocrystals: the PP molecule in PC_PP is green, the PP molecule in MC_PP is yellow, the PP molecule in OC_PP is red and the single free PP molecule is black. Compared with the red, yellow and black structure, the green structure shows more obvious deformation (units: kcal mol^−1^). Surface local minima and maxima of ESP are represented as cyan and orange spheres, respectively. The global minimum and maximum values are italic.

The consistency and variability of cocrystals containing the positional isomers of MC_PP, OC_PP and PC_PP have been demonstrated using the crystal structures, HS analysis, spectral analysis and interaction energies. However, the reasons for consistency and variability are not particularly clear from the above analysis, although we can see that all weak interactions are related to hydrogen bonds of different strengths. IUPAC redefines hydrogen bonding, suggesting that the formation of a hydrogen bond is primarily an electrostatic interaction resulting from charge transfer between the donor and acceptor. Hence, the hydrogen bond strength is strongly correlated to the extent of charge transfer. The greater the charge transfer between molecules, the stronger the covalency of the hydrogen bond (Arunanl *et al.*, 2011 ▸; Zhang *et al.*, 2013*a* ▸,*b* ▸). In 2011, Bahers *et al.* (2011 ▸) proposed a method for analysing charge transfer during electron transitions and this method can also be used to study charge transfer in the formation of a molecular complex (Zhu *et al.*, 2015 ▸; Lu & Chen, 2012*c* ▸). In order to determine the reason for consistency and variability of the cocrystals formed between the three cresol isomers (MC, OC and PC) and the same coformer (PP molecule), the charge transfer between the fragments of two molecules was computed using ADCH charges in the gas phase; the results are shown in Table 3 ▸. For hydrogen bonding, the molecules tend to contact each other in an electrostatically complementary manner to maximize electrostatic interaction and reduce the energy of the system. Also, MESPs on molecular vdW surfaces have played a major role in elucidating the nature of these intermolecular electrostatic interactions (Murray & Politzer, 2011 ▸, 2017 ▸; Lu & Chen, 2012*c* ▸). Therefore, to reveal the consistency and variability of the cresol cocrystals, the MESPs of monomer and complex molecules, together with the deformation of the PP molecule, were also studied and the results are shown in Fig. 6 ▸. It can be seen from Table 3 ▸ that the extent of charge transfer for O—H⋯N hydrogen bonds is significantly larger than that of π⋯H, which indicates that the O—H⋯N hydrogen bond has a stronger effect. In addition, the charge transfer between the MC/OC molecule and the PP molecule is almost the same, −0.131241 a.u. and −0.132840 a.u., respectively. Atomic dipole moment corrected Hirshfeld (ADCH) charges are transferred from one MC and/or OC molecule to the PP molecule *via* O—H⋯N hydrogen bonds (Lu & Chen, 2012*c* ▸). However, the charge transfer between PC and PP molecules is higher than that between MC_PP and OC_PP synthons, approximately 0.004–0.005 a.u. ADCH charges transferred. This could be one reason for the consistency and variability of the cocrystals.

The quantitative molecular surface analysis module of the *Multiwfn* program (Lu & Chen., 2012*a* ▸,*c* ▸) is capable of partitioning the whole vdW surface into multiple fragments, allowing us to study the characteristics of electrostatic potential distribution (Lu & Manzetti, 2014 ▸). Although HS is also a molecular surface, it can only reflect the weak interactions between adjacent molecules in a molecular crystal. Hence, HS can only reflect the role of the crystal in the results and cannot fully show the state prior to crystal formation. Moreover, the analyses of the structure (mainly bond lengths) as well as the spectral and interaction energies show that the O—H⋯N hydrogen bond plays a dominant role in the cocrystal formation, which is also the main factor that results in consistency and variability. Therefore, in order to find the essential cause of the consistency and variability when performing the MESP analysis, only the sites formed by O—H⋯N hydrogen bonds were considered; the results are given in Fig. 6 ▸.

Fig. 6 ▸ shows that for the three cresol isomers (OC, MC, PC), the most positive electrostatic potential (ESP) value (global maximum ESP value) on the vdW surface is at the position of the hydroxyl hydrogen (OC: +51.93, MC: +51.22, PC: +51.02 kcal mol^−1^), while there is an obvious negative potential at the benzene ring and the lone pair of electrons on the oxygen atoms. The values of the most negative potentials (the global maximum) are −25.25 kcal mol^−1^ for OC, −27.83 kcal mol^−1^ for MC and −28.20 kcal mol^−1^ for PC, stemming from the prominent lone pair of electrons on O. Hydroxyl groups can serve as donors and acceptors for hydrogen bonds, and the most negative ESP value on the PP vdW surface is −35.81 kcal mol^−1^, stemming from the prominent lone pair of electrons on N of the imino group, whereas the most positive ESP value is +24.93 kcal mol^−1^ at the position of the H on the imino group. According to the principle of complementary electrostatic potential, compared with the interaction between the same molecules (PP–PP or cresol–cresol), cresol isomer molecules are highly compatible with the electrostatic potential of the PP molecule to reduce the energy of the system, which makes it easier to form heterosynthons, in which, the cresol molecules act as the hydrogen bond donors while the PP molecules act as the hydrogen bond acceptors. This is consistent with the experimental results. However, why do the three cocrystals formed between cresol isomers and PP molecules exhibit very obvious consistency and variability? As the hydrogen bond donor, the most positive ESP value of PC molecule is the minimum among these three cresol isomers. Although the maximum positive ESP of PC is only 0.2 kcal mol^−1^ less than that of MC, we believe that the maximum positive ESP on MC is the lowest limitation for the cocrystal formation with a stoichiometric ratio of 2:1, and a smaller positive ESP than the positive ESP of MC does not have enough power to bind another imino group on the same PP molecule. In addition, the MESPs of the synthons of only one cresol molecule with one PP molecule are also exhibited in Fig. 6 ▸. The negative potential (−30.68 kcal mol^−1^) of the other end of the same PP molecule (unoccupied end) in the PC_PP synthon is significantly lower than that of a single PP molecule (−35.81 kcal mol^−1^). Therefore, the structure and the electrostatic potential are detrimental to the binding of the second PC molecule. Whereas for MC_PP and OC_PP synthons, the positive potential on the unoccupied end of the same PP molecule decreases less (−32.16 kcal mol^−1^ and/or from −31.76 to −35.81 kcal mol^−1^, respectively) and the positive potentials of MC and OC are larger than that of PC. Therefore, they have the ability to form a synthon with a ratio of 2:1. The binding mode also results in the elongation of PP molecules in MC_PP and OC_PP cocrystals, as shown in Fig. 6 ▸(*h*). Moreover, the reason why the homosynthon formed with N—H⋯N hydrogen bonds can be formed in PC_PP but not in MC_PP or OC_PP can also be found from the MESP maps. Although the portion where the hydrogen bonding is formed causes the global maximum and minimum values (−35.17 and +35.19 kcal mol^−1^ for MC1PP, and −32.02 and +35.67 kcal mol^−1^ for OC1PP) of the ESP, there is a negative local minimum point (negative potential, −2.75 kcal mol^−1^ for MC1PP and −2.03 kcal mol^−1^ for OC1PP) near the global maximum values (positive potential) of MC1PP and OC1PP, which is unfavorable for the imino group, as hydrogen bond donor, to form hydrogen bonds with a PP molecule. The deformation degree of the PP molecule in the MC1PP and OC1PP synthons is not significant, resulting in large steric hindrance and hence inhibition of the binding of another PP molecule to form a homosynthon. However, this negative local minimum point does not appear for PC_PP, so it can be combined with another PP molecule to form a homosynthon.

Since the essence of hydrogen bonding is electrostatic interaction, the reason for the consistency and variability in the cresol–piperazine cocrystal system can be inferred from the extent of charge transfer and the quantitative MESP analysis.

## 3. Conclusions   

The consistency and variability of cocrystals containing positional isomers were investigated by combined experimental and theoretical approaches using cresol isomers and piperazine as model compounds. From analysing the structures of the crystals, supramolecular synthons and LSAMs, the nature of weak interctions and atomic charge, the consistency and variability of cocrystals containing positional isomers were analyzed and discussed. Although all three isomers of cresol can form corresponding cocrystals with PP molecules, the obtained cocrystals exhibit some consistency and variability in crystal structure, stoichiometric ratio, synthon pattern, weak interactions, LSAMs, interaction energies, physical properties and self-assembly mechanisms. It also shows that PP molecules have different affinities for the selective combination of cresol isomer molecules to form a cocrystal. The molecular recognition and self-assembly mechanism of supramolecular synthons of cresol–piperazine in toluene solution and its evolution pathway was investigated by means of spectroscopy, PAT and theoretical calculations. We found that the formation of these three cocrystals can be divided into three steps: (i) heterotrimer or heterodimer formation, (ii) cocrystal nucleation and (iii) cocrystal growth. Thus, we propose that the supramolecular synthons are firstly formed in solution prior to the formation of the solid cocrystal and the synthon structures formed initially in solution carry over into the final product. The supramolecular synthon structures are the precursors of the cocrystals and the information memory for the cocrystallization process. Furthermore, the MESP was quantitatively analyzed using DFT theory, and the reasons for consistency and variability were found by principles of electrostatic potential complementation. In the cresol–piperazine cocrystal system, as the hydrogen bond donor, the global maximum positive potential of the PC molecule is the lowest compared with MC and OC molecules. Although PC is only 0.2 kcal mol^−1^ lower in energy than MC, the MC molecule is the bottom limit of the cocrystal formation with the PP molecule, which is essentially the cause of the consistency and variability of the cresol–piperazine cocrystals. Quantitative analysis of the MESP will be helpful for guiding screening of cocrystals.

## 4. Related literature   

The following references are cited in the supporting information: Spackman & Jayatilaka (2009 ▸); Spackman & McKinnon (2008 ▸); Ravat *et al.* (2015 ▸); Spackman & Byrom (1997 ▸); Mckinnon *et al.* (2004 ▸); Zhao & Truhlar (2008*a* ▸,*b* ▸,*c* ▸, 2011 ▸); Grimme *et al.* (2010 ▸); Frisch *et al.* (2009 ▸); Antony *et al.* (2015 ▸); Boys & Bernardi (2002 ▸); Marenich *et al.* (2009 ▸); Tomasi *et al.* (2005 ▸); Alecu *et al.* (2010 ▸); Li *et al.* (2018 ▸); Lu & Chen (2012*b* ▸); Humphrey *et al.* (1996 ▸); Turner *et al.* (2017 ▸).

## Supplementary Material

Crystal structure: contains datablock(s) global, MC_PP, OC_PP, PC_PP. DOI: 10.1107/S2052252519012363/lq5023sup1.cif

Structure factors: contains datablock(s) MC_PP. DOI: 10.1107/S2052252519012363/lq5023MC_PPsup2.hkl

Structure factors: contains datablock(s) OC_PP. DOI: 10.1107/S2052252519012363/lq5023OC_PPsup3.hkl

Structure factors: contains datablock(s) PC_PP. DOI: 10.1107/S2052252519012363/lq5023PC_PPsup4.hkl

Supporting information file. DOI: 10.1107/S2052252519012363/lq5023sup5.pdf

CCDC references: 1906693, 1907006, 1907017

## Figures and Tables

**Table 1:** Supramolecular synthon and hydrogen bond information

D—H⋯A			*d*(D—H)	*d*(H⋯A)	*d*(D⋯A)	∠(DHA)	symop_for_A
MC_PP	O(1)—H(1D)⋯N(1)	Exp.†	0.840	1.860	2.679 (2)	165	*x*−1/2, −*y*+1/2, −*z*+1
		Calc.‡	0.995	1.746	2.726	168	–
	N(1)—H(1)⋯π	Exp.	–	2.458§	–	–	–
		Calc.	–	2.344§	–	–	–
OC_PP	O(1)—H(1 A)⋯N(1)	Exp.	0.840	1.887	2.715 (1)	169	1+*x*, *y*, *z*
		Calc.	0.995	1.746	2.726	168	–
	N(1)—H(1)⋯π	Exp.	–	2.442§	–	–	–
		Calc.	–	2.392§	–	–	–
PC_PP	O(1)—H(1D)⋯N(1)	Exp.	0.87 (1)	1.83 (2)	2.684 (1)	167 (1)	*x*, −1+*y*, *z*
		Calc.	0.995	1.772	2.757	170	–
	N(1)-H(1)⋯N(2)	Exp.	0.90 (1)	2.15 (1)	3.038 (1)	170 (1)	2−*x*, −1/2+*y*, 1/2−*z*
		Calc.	1.020	2.146	3.164	175	–
	N(2)—H(2)⋯π	Exp.	–	2.431§	–	–	–
		Calc.	–	2.359§	–	–	–

† Weak interaction results of single-crystal structures.

‡ Weak interaction results of optimized structures.

§ Hydrogen bond length between the donor hydrogen and the center of the benzene ring.

**Table 2:** Intermolecular interaction energies of synthons

			Gas phase		Toluene		
Cocrystals	Synthons		Δ*E*_t_ † (kcal mol^−1^)	Δ*E*_d_ ‡ (kcal mol^−1^)	Δ*E*_t_ (kcal mol^−1^)	Δ*E*_d_ (kcal mol^−1^)	Lattice energy (kcal mol^−1^)
MC_PP	I	O(1)—H(1D)⋯N(1)	−22.53	−11.27	−21.26	−10.63	39.65
	II	N(1)—H(1)⋯π	−12.96	−6.48	−10.15	−5.08	
OC_PP	I	O(1)—H(1 A)⋯N(1)	−22.64	−11.32	−21.34	−10.67	40.91
	II	N(1)—H(1)⋯π	−12.59	−6.30	−9.75	−4.87	
PC_PP	I	O(1)—H(1D)⋯N(1)	–	−13.51	–	−12.17	83.41
	II	N(1)—H(1)⋯π	–	−7.38	–	−5.95	
	III	N(1)—H(1)⋯N(2)	–	−8.65	–	−7.14	

† Intermolecular interaction energy of the synthon.

‡ Average intermolecular interaction energy of the synthons: Δ*E*_d_ = Δ*E*_t_/(total number of interactions).

**Table 3:** Charge transfer between molecules of the cocrystals

Complex	ADCH (G) (a.u.)		
	(*m*-, *o*-, *p*-)C→PP (hydrogen bonding)	(*m*-, *o*-, *p*-)C→PP (π⋯H)	PP→PP (hydrogen bonding)
MC_PP	−0.131241	0.072469	–
OC_PP	−0.132840	0.073676	–
PC_PP	−0.136968	0.068073	−0.065889
