# Supplementary material for: Consistency and variability of cocrystals containing positional isomers: the self-assembly evolution mechanism of supramolecular synthons of cresol–piperazine
Source: IUCrJ. 2019 Oct 9;6(Pt 6):1064–73. doi: 10.1107/S2052252519012363 (PMC6830220; doi:10.1107/S2052252519012363)
Supplement: Supplementary file 5 [file m-06-01064-sup5.pdf]

IUCrJ

Volume 6 (2019)

Supporting information for article:

Consistency and variability of positional isomerism cocrystals: self-assembly evolution mechanism of supramolecular synthons of cresol-piperazine

Na Wang, Xin Huang, Lihang Chen, Jinyue Yang, Xin Li, Jiayuan Ma, Ying Bao, Fei Li, Qiuxiang Yin and Hongxun Hao

S1. Experimental Section

S1.1. Materials and analytical tools

Three isomers of cresol, *m*-, *o*- and *p*-cresol (mass fraction: w% > 99.0%, abbreviated as MC, OC and PC in the text, respectively) were purchased from Tianjin Yuanli Chemical Co., Ltd. The analytical-grade piperazine (PP) and toluene were in-sourced in the Tianjin Guangfu Fine Chemical Research Institute. The toluene-d8 (Toluene-d8, 99.9 atom % D, contains 0.03% (v/v) TMS) was purchased from SIGMA-ALORICH Co., of USA. All chemicals were used without further purification. **Figure S1** shows the molecular structures of starting materials and **Table S1** gives the basic information of cresol isomers and piperazine.

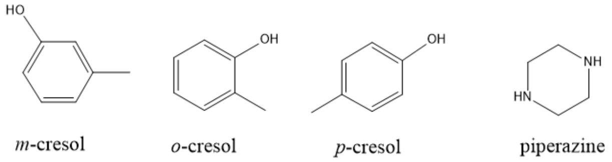

Figure S1 Molecular structures of (*m*-, *o*-, *p*-) cresol and piperazine.

Table S1 Basic information of piperazine and cresol isomers [a]

	Cresol isomers			Coformer
	o-cresol (OC)	*m*-cresol (MC)	*p*-cresol (PC)	piperazine [b] (PP)
Molecular Weight (g/mol)	108.14			86.14
Melting Point (K)	30.94 [c]	11.95 [c]	34.78 [c]	109.6 [b]
Boiling Point (1 atm) (K)	190.95	202.2	201.9	148.5 [b]

[a] Editorial board of <Handbook of Applied Chemistry>. Handbook of practical chemistry. Beijing: Science Press, 2001.

[b] Cheng, N. L. Solvent Handbook (4th edition). Beijing: Chemical Industry Press (CIP), 2007:504-512.

[c] http://www.somsds.com/detail.asp?id=12285

S1.1.1. X-ray diffraction

The powder X-ray diffraction (PXRD) patterns were collected by Rigaku D/MAX 2500 in 2θ range from 2° to 50°, with a step size of 0.02°, current of 100 mA and voltage of 40 kV. And the data collection for the single cocrystals were carried out on a Rigaku-Rapid II diffractometer with Mercury2 CCD area-detector by using graphite-monochromatized Mo Kα radiation at a wavelength of 0.71073 Å.

S1.1.2. Thermal analysis

The thermodynamic properties of the cocrystals of cresol isomers and PP were determined using Mettler-Toledo DSC 1/500 instrument under protection of nitrogen gas with purging rate of 50 mL/min. And the measurement temperature range was 25-105 °C with heating rate of 5 °C /min.

S1.1.3. ^{1}H-nuclear magnetic resonance (^{1}H NMR)

^{1}H NMR analyses were performed on a Varian Inova 500 MHz Spectrometer (Palo Alto, CA, USA). Approximately 15~20 mg samples were dissolved into 0.6 ml of toluene-d8. In fact, 16.5 mg of **MC_PP** cocrystal, 17.6 mg of **OC_PP** cocrystal, 16.6 mg of **PC_PP** cocrystal, 15.2 mg of MC, 13.5 mg of OC, 12.1 mg of PC and 18.6 mg of PP were dissolved into 0.6 ml of toluene-d8, respectively. All spectra were collected at 298.15 K. And the chemical shifts are referenced to internal TMS (0.00 ppm)

S1.1.4. Fourier transformed infrared spectrometer (FTIR)

Infrared spectra of cocrystals and their constituents were collected from 400 to 4000 cm^{-1}, using the ALPHA II infrared instrument (BRUKER, Germany). And the spectra of monomers or dimers or trimers in solution were recorded from 650 to 2800 cm^{-1}. The formation of the dimers or trimers were monitored by **Attenuated Total Reflectance Fourier transformed infrared spectroscopy (ATR-FTIR)**. The interactions between cresol isomers and PP were confirmed based on the wavenumbers.

S1.1.5. Raman spectroscopy (Raman)

The ***RamanRXN2™ HYBRID*** analyser (Kaiser Optical Systems, Inc. USA) was used for on-line measurements of the cocrystals formation in solution. The Raman spectra were record from 300 to 1890 cm^{-1}. The *PhAT* probe was used to collect the characteristic peaks for qualitative analysis while the *MR* probe was used to *in-situ* detect the formation of the cocrystals in solution during the cocrystallization process.

S2. Preparation of single cocrystals and PAT monitoring of cocrystallization processes

The same crystallization method was used for the three cocrystals. The best cocrystals were obtained from slow room-temperature evaporation of toluene at the ratio of 1:1 or 1:2 of pure compound.

The PAT tools were used to *in-situ* monitor cocrystallization processes of the three cocrystals in slow cooling crystallization in toluene, and the experimental conditions are shown in **Figure S2**. All experiments were performed in a 110 mL double-jacketed glass crystallizer with an overhead mechanical agitator to mix the solution, and the temperature of jacketed crystallizer was controlled by a thermostat with temperature accuracy of ±0.01 °C. The initial material ratios of all experiments are shown in **Figure S2**. And at these ratios of materials, a clear solution can be obtained at the higher temperature (40 °C for MC + PP, 38 °C for OC + PP and 70 °C for PC + PP, respectively) in all

experiments. After stabilizing at the initial high temperature for a period of time, the temperature of all systems was decreased to the final experimental temperature at a cooling rate of 0.05 °C /min. PAT monitoring will be stopped after these systems reaches stability at the final temperature. Additionally, when sufficient supersaturation is achieved at certain temperature (37.5 °C for MC + PP and 31 °C for OC + PP, respectively), about 0.05g crystal seeds will be added to the crystallizer to induce nucleation, except for the "PC + PP" experiment. During these experiments, Raman and ATR-FTIR spectroscopic analysers were applied in combination to *in-situ* monitor the formation process of (*m*-, *o*-, *p*-) cresol-piperazine cocrystals. The final products were also analyzed by PXRD, DSC, FTIR, Raman and ^{1}H NMR.

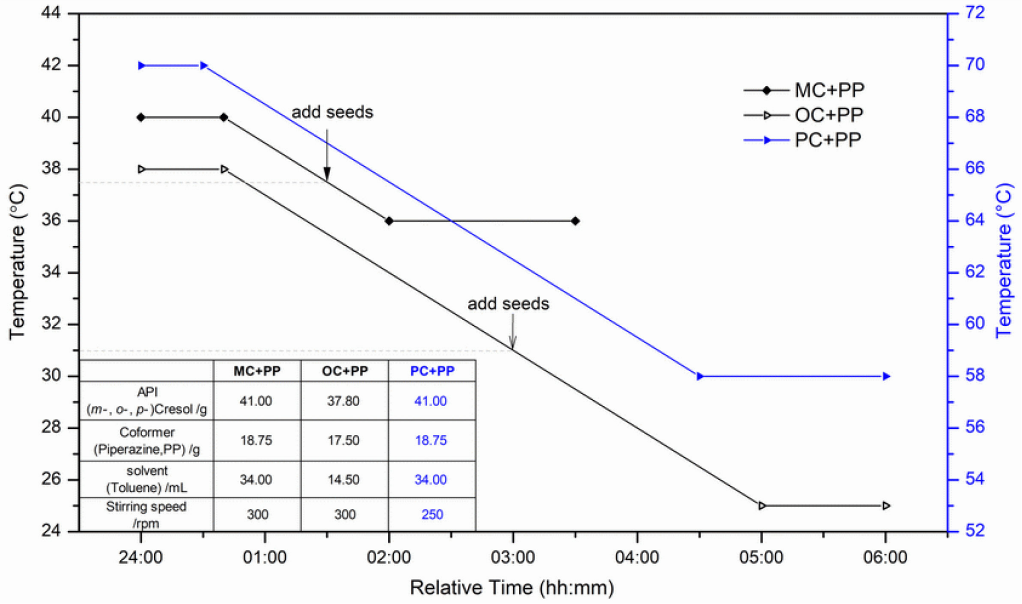

Figure S2 The experimental conditions for the cocrystallization process monitored by PAT tools.

S3. Results & Discussion

S3.1. Crystal structures and structural consistency and variability

S3.1.1. Crystal structures and molecular arrangements

In **MC_PP** cocrystal, obtained by cocrystallizing PP and MC molecules, PP molecules occupy all the eight vertices and all six face-centers of the cuboid cell, while MC molecules fill in the void positions in the unit cell, as shown in **Figure 1a)**. The PP molecules mainly distributes on the XY-plane (or OAB-plane), and two layers of MC molecules (two yellow molecular layers) with opposite stacking direction, such as the filling of sandwich biscuit, are filled between the two opposite PP molecular chains (two green molecular chains), as shown in **Figure 1b)**. Along the *z*-axis (or *oc*-axis), two adjacent MC molecules layers interacting with different PP molecules layers are arranged in a herringbone-type fashion (the yellow sticky molecules), while the two MC molecules interacting with the same PP molecule are on two mutually parallel planes, connected by strong hydrogen bonding

(O(1)-H(1D)···N(1)), as illustrated in **Figure 1c)**, marked as **Synthons I** under light purple shadow. In adjacent two MC molecules layers along the *y*-axis (or *ob*-axis), the MC molecules are arranged in a cross-wise arrangement and show an interlocked structure, as shown in **Figure 1b)**. In addition, two types of supramolecular synthons composed of one PP molecule and two MC molecules are formed: For **Synthon I** (as shown in **Figure 1c)**, under light purple shadow), PP molecule (molecule 2) is the symmetric center and two MC molecules (molecule 1 and 1') symmetrically distributed in the PP center on two parallel planes, mainly connected by strong hydrogen bonding (O(1)-H(1D)···N(1)), named as **MC_PP**. These three molecules are not on the same plane, but form three steps. For **Synthon II** (as shown in **Figure 1c)**, under light green shadow), PP molecule (molecule 2) is also the symmetric center and the two MC molecules (molecule 1 and 1') are distributed in the PP center on another two parallel planes, mainly connected by π···H hydrogen bonding (N(1)-H(1)···π), named as **PI_MC_PP**. And the angle between the two supramolecular synthons is about 90°, as shown in **Figure 1c)**, and the two primary synthons interacted by O(1)-H(1D)···N(1) and N(1)-H(1)···π assemble the one-dimension LSAM (LSAM (1D)), as shown in **Figure 1c)**. The LSAMs (1D) assemble into corrugated close-packing two-dimension LSAM (LSAM (2D)), as shown in **Figure 1d)**. And the 2D LSAMs are symmetrically inverted V-shape. There is no obvious interaction between the parallel MC molecules (molecules 1 and 1, or molecules 1' and 1'), so is the parallel PP molecules (molecules 2 and 2). As shown in **Figure 1c)** and **d)**, the three molecular chains consisting of molecules MC (1), molecules MC (1') and molecules PP (2) respectively, are parallel to each other. Moreover, four MC molecules perfectly surround the PP molecule, preventing the interaction between the second PP molecule with this PP molecule. The types of supramolecular synthons and intermolecular interaction are summarized in **Table 1**, and all the intermolecular interactions analyzed in this part are also verified by the following Hirshfeld surface (HS) analysis.

In the cocrystal of **OC_PP** formed by one PP molecule and two OC molecules, the crystal structure, the packing model and supramolecular synthons are almost identical to **MC_PP** cocrystal, although they belong to different crystal systems (**OC_PP** cocrystal belonging to monoclinic system and **MC_PP** cocrystal belonging to orthogonal system, respectively), as shown in **Table S2** and **Figure S3**. PP molecules occupy all the eight vertices and body-center of the cuboid cell, while OC molecules fill in the void positions in the unit cell, as shown in **Figure S3a)**. There are also two types of vertically arranged supramolecular synthons, composed of one PP molecule and two OC molecules, and mainly connected by two different types of hydrogen bonding (O(1)-H(1A)···N(1), and N(1)-H(1)···π).

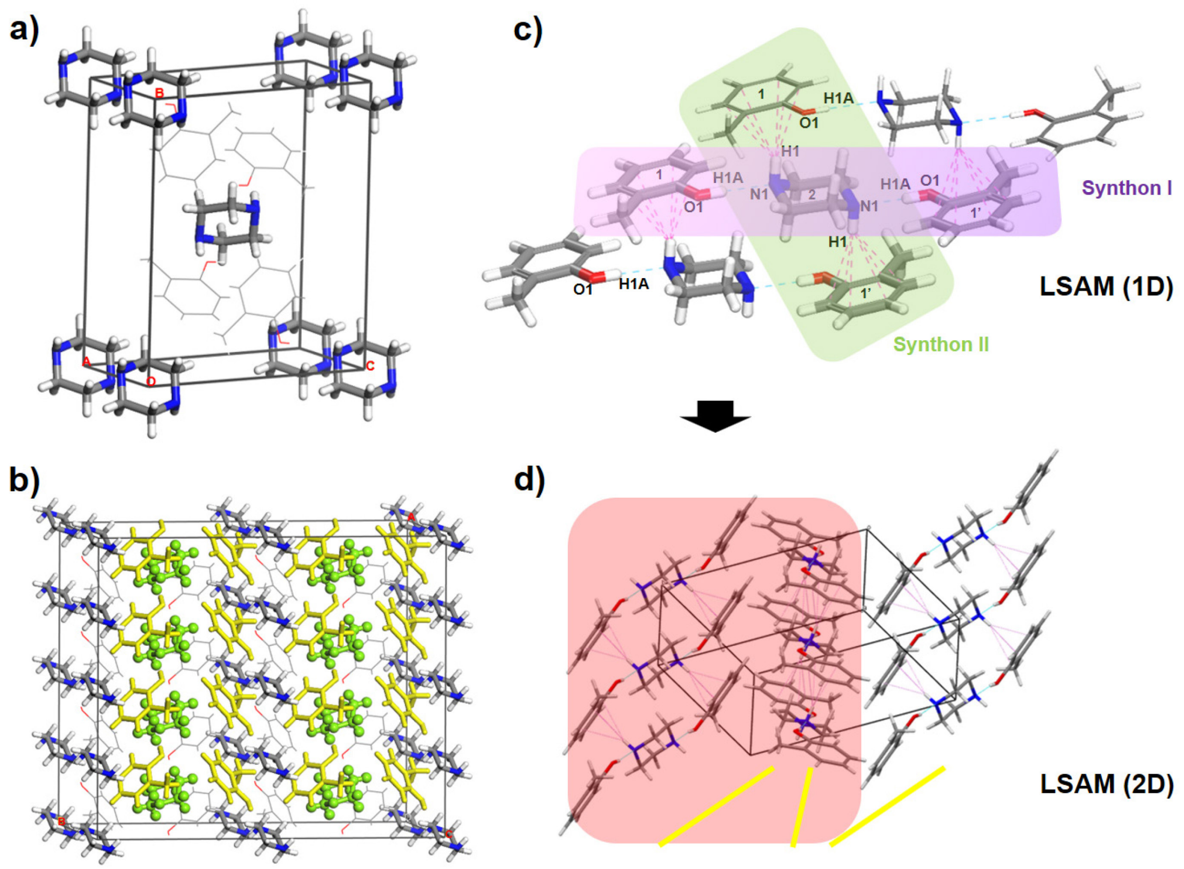

Figure S3 The crystal structure, packing model and intermolecular interactions of **OC_PP** cocrystal. a) the unit cell of **OC_PP** cocrystal. b) the 3D supramolecular packing model in supercell with 4×2×1. c) LSAM (1D) constructed by amalgamation of **Synthon I** (supramolecular synthon under light purple shadow interacted by O-H···N hydrogen bonding) and **Synthon II** (supramolecular synthon under light green shadow interacted by N-H···π hydrogen bonding). d) two-dimension LSAM (LSAM (2D)) structure (under light red shadow) constructed by arrangement of the LSAMs (1D). Purple dotted lines represent π···H hydrogen bonding, while blue dotted lines represent O-H···N hydrogen bonding.

Table S2 Crystallographic information for the cocrystals

	**MC_PP** cocrystal	**OC_PP** cocrystal	**PC_PP** cocrystal
Crystal data			
Chemical formula	$C_{18}H_{26}N_2O_2$	$C_{18}H_{26}N_2O_2$	$C_{11}H_{18}N_2O$
M_r	302.41	302.41	194.27
Crystal system, space group	Orthorhombic, *Pbca*	Monoclinic, $P2_1/n$	Orthorhombic, $P2_12_12_1$
Temperature (K)	113	133	113
a, *b*, *c* (Å)	5.5482 (17), 12.167 (3), 24.556 (6)	5.6352 (8), 13.4281 (17), 11.2825 (15)	5.9035 (10), 8.3771 (16), 21.980 (4)
β (°)		93.703 (3)	
V (Å^3)	1657.6 (8)	852.0 (2)	1087.0 (3)
Z	4	2	4
Radiation type	Mo *K*α	Mo *K*α	Mo *K*α
μ (mm^{-1})	0.08	0.08	0.08
Crystal size (mm)	0.20 × 0.18 × 0.12	0.20 × 0.18 × 0.12	0.20 × 0.18 × 0.12
Data collection			
Diffractometer	Rigaku XtaLAB P200		
Absorption correction	Multi-scan REQAB (Rigaku, 1998)		
T_{min}, T_{max}	0.984, 0.991	0.985, 0.991	0.985, 0.991
No. of measured, independent and observed [$I > 2\sigma(I)$] reflections	14815, 1882, 1509	8549, 1950, 1756	13953, 2501, 2450
R_{int}	0.053	0.038	0.015
(sin θ/λ)$_{max}$ (Å^{-1})	0.651	0.651	0.650
Refinement			
$R[F^2 > 2\sigma(F^2)]$, $wR(F^2)$, S	0.044, 0.124, 1.06	0.041, 0.126, 1.07	0.027, 0.076, 1.07
No. of reflections	1882	1950	2501
No. of parameters	105	106	140
No. of restraints	1	1	3
H-atom treatment	H atoms treated by a mixture of independent and constrained refinement		
$\Delta\rho_{max}$, $\Delta\rho_{min}$ (e Å^{-3})	0.27, −0.19	0.26, −0.24	0.19, −0.17

Computer programs: *CrystalClear*-SM Expert 2.1 b42 (Rigaku, 2015), *CrystalClear*-SM Expert 2.1 b42, *SHELXS97* (Sheldrick, 1990), *SHELXL97* (Sheldrick, 1997), *CrystalStructure* 4.1 (Rigaku, 2014).

In **OC_PP** cocrystal, PP molecules occupy all the eight vertices and body-center of the cuboid cell, while OC molecules fill in the void positions in the unit cell, as shown in **Figure S3a)**. The PP molecules mainly distribute on the XZ-plane (or OAC-plane), and only one layer of OC molecules

(one yellow molecular layer), such as the filling of sandwich biscuit, are filled between the two opposite PP molecular chains (one PP molecular chain with green highlighted and one PP molecular chain without thickened and highlighted), as shown in **Figure S3b)**. In adjacent two OC molecules layers along the *z*-axis (or *oc*-axis), the molecules in different layers are not arranged in parallel, but arranged with a certain dihedral angle, as shown in **Figure S3b)**. In addition, there are also two types of supramolecular synthons, composed of one PP molecule and two OC molecules: For **Synthon I** (under light purple shadow), the PP molecule (molecule 2) is the symmetric center and two OC molecules (molecule 1 and 1') symmetrically distributed in the PP center on two parallel planes, mainly connected by strong hydrogen bonding (O(1)-H(1A)···N(1)), named as **OC_PP**, as shown in **Figure S3c**. These three molecules are not on the same plane, but form three steps. For **Synthon II** (under light green shadow), PP molecule (molecule 2) is also the symmetric center and two OC molecules (molecule 1 and 1') are distributed in the PP center on two parallel planes, mainly connected by π···H hydrogen bonding (N(1)-H(1)···π), named as **PI_OC_PP**, as shown in **Figure S3c**. The angle between the two supramolecular synthons is almost about 90°, as shown in **Figure S3c)**, and the two primary synthons interacted by O(1)-H(1A)···N(1) and N(1)-H(1)···π assemble the one-dimension LSAM (LSAM (1D)), as shown in **Figure S3c)**. The LSAMs (1D) assemble into corrugated close-packing two-dimension LSAM (LSAM (2D)), as shown in **Figure S3d)**, and the 2D LSAMs are in a twisted inverted V-shape. There is no obvious interaction between the parallel OC molecules (molecules 1 and 1, or molecules 1' and 1'), so is the parallel PP molecules (molecules 2 and 2). As shown in **Figure S3c)** and **d)**, the three molecular chains consisting of molecules OC (1), molecules OC (1') and molecules PP (2) respectively, are parallel to each other. Four OC molecules perfectly surround the PP molecule, preventing the interaction between the second PP molecule with this PP molecule. The types of supramolecular synthons and intermolecular interaction are summarized in **Table 1**.

In the unit cell of 1:1 P**C_PP** cocrystal, all the molecules are packed into the cell of the cuboid, and no molecules occupy the feature location, *e.g.* vertices, body-centers and face-centers, as shown in **Figure S4a)**. The PP molecules mainly distributes on the XY-plane (or OAB-plane), and two layers of PC molecules (one yellow sticky PC layer and one ball-stick PC molecule layer), like the filling of sandwich biscuit, are filled between the two opposite PP molecular chains (two green PP molecular chain with highlighted), as shown in **Figure S4b)**. The PP molecules are connected end to end along the *y*-axis (or *ob*-axis), forming a corrugated chain, which runs through the entire crystal, as shown in **Figure S4b)** and **S4d)**, and the PC molecules are connected at the bend of the zigzag chains, vertical to the PP molecule, as shown in **Figure S4c)**. On the same PP corrugated chain formed by N(1)-H(1)···N(2) between two different PP molecules, one PC molecule (molecule 1) and two mutually parallel but unconnected PP molecules (molecule 2 and 3) interact by hydrogen bonding O(1)-H(1D)···N(1) and N(2)-H(2)···π, respectively, as shown in **Figure S4c)** and **S4d)**. Nevertheless, there

is no apparent and direct weak interaction between two adjacent (molecule 1 and 1') and/or parallel (molecule 1 and 1) PC molecules, as shown in **Figure S4c)**. Two adjacent PP chains has no other strong interaction except for van der Waals interaction. In two adjacent PC molecules layers along the *z*-axis (or *oc*-axis), the molecules in different layers between the two PP chains are not arranged in parallel, but arranged with a certain dihedral angle, as shown in **Figure S4b)**.

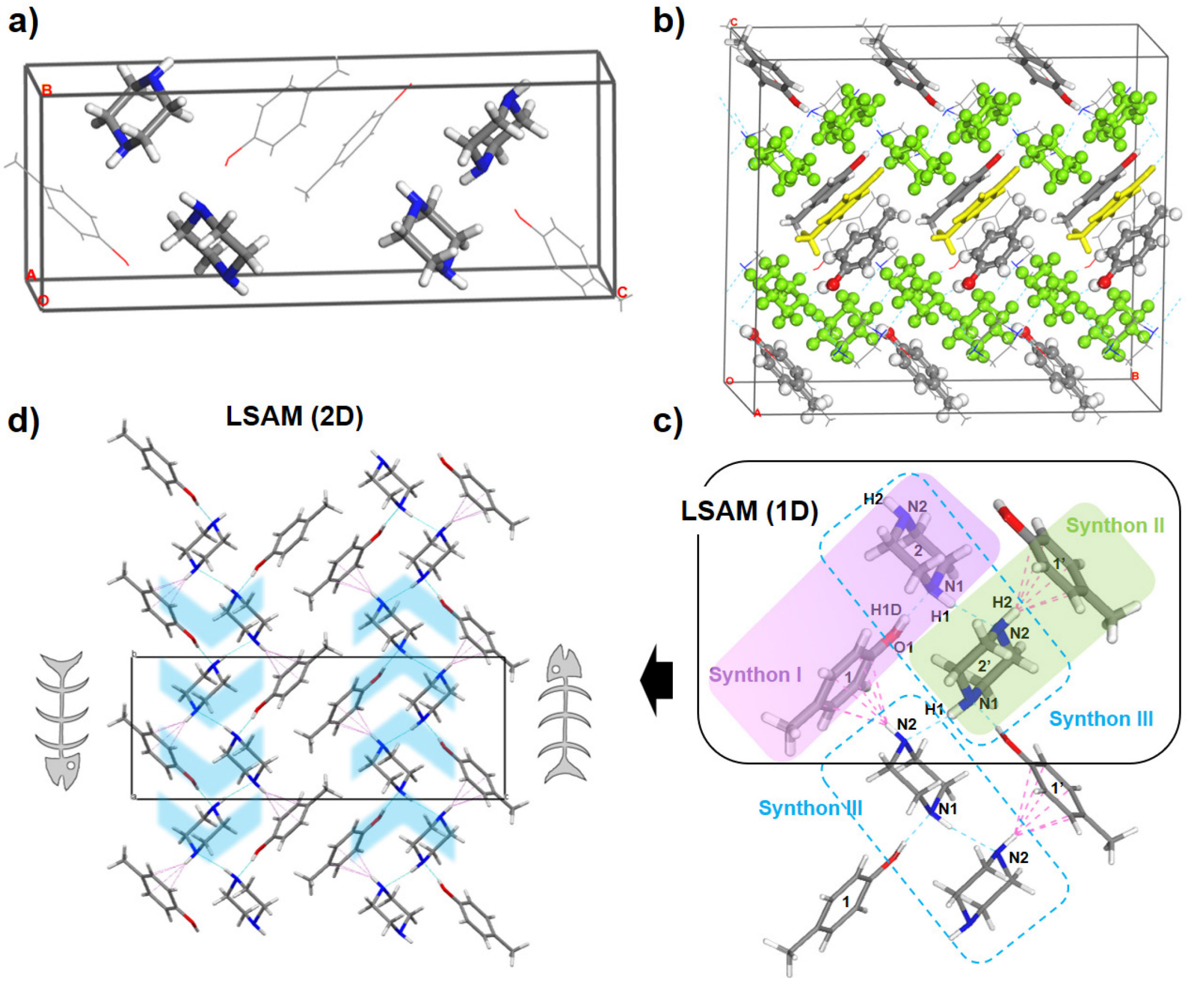

Figure S4 The crystal structure, packing model and intermolecular interactions of **PC_PP** cocrystal. a) the unit cell of **PC_PP** cocrystal. b) the 3D supramolecular packing model in supercell with 2×3×1. c) LSAM (1D) constructed by amalgamation of **Synthon I** (supramolecular synthon under light purple shadow interacted by O-H···N hydrogen bonding), **Synthon II** (supramolecular synthon under light green shadow interacted by N-H···π hydrogen bonding) and **Synthon III** (supramolecular synthon inside the blue dotted frame interacted by N-H···N hydrogen bonding). d) two-dimension LSAM (LSAM (2D)) structure constructed by arrangement of the LSAMs (1D), two corrugated PP molecules chains form two herringbone-type fashion in opposite direction. Purple dotted lines represent π···H hydrogen bonding, while blue dotted lines represent O-H···N and N-H···N hydrogen bonding.

The structural features and conformations of **PC_PP** cocrystal are apparently different from **OC_PP** cocrystal and **MC_PP** cocrystal and so are the interactions. The asymmetric unit of **PC_PP** cocrystal contains one PP molecule and one PC molecule, as shown in **Table S2** and **Figure S4**. Particularly, it is worth noting that there are three different supramolecular synthon modes in **PC_PP** cocrystal compared to the **MC_PP** and **OC_PP** cocrystals: expect for the two heterosynthons formed by PP and PC molecules interacted with O(1)-H(1D)···N(1) (named as **PC_PP**, **synthon I**) and the N(2)-H(2)···π (named as **PI_PC_PP**, **synthon II**), respectively, another homosynthon is also formed by two PP molecules interacted with N(1)-H(1)···N(2), named as **PP2** (**synthon III**), as shown in **Table 1** and **Figure S4**. Another unique feature is that the PP molecules are connected end to end, forming a corrugated chain, which runs through the entire crystal. And no molecules occupy the vertices, face-center and body-center of the cuboid unit cell.

What is more, different from the **MC_PP** and **OC_PP** cocrystals, three types of supramolecular synthons are formed: including two heterosynthons composed of one PP molecule and one PC molecule, and one homosynthon formed by two PP molecules. Both the two heterosynthons are formed between the PP molecule and the PC molecule, and interactions occur at different positions of imino-group on the same PP molecule: **Synthon I** is mainly connected by strong hydrogen bonding (O(1)-H(1D)···N(1)), named as **PC_PP**, with the hydroxyl hydrogen on the PC molecule acting as hydrogen bonding donor and the N atom on the PP molecule acting as the hydrogen bonding acceptor, as shown in **Figure S4c)** (under light purple shadow). The **Synthon III** is mainly connected by N(1)-H(1)···π hydrogen bonding, named as **PI_PC_PP**, and the H atom on the imino-group of PP molecule acts as the hydrogen bonding donor and the benzene ring acts as the hydrogen bonding acceptor, as shown in **Figure S4c)** (under light blue shadow). The homosynthon is consisted of two PP molecules and connected by N(1)-H(1)···N(2) hydrogen bonding, named as **PP2**, as shown in **Figure S4c)** (**Synthon III**, inside blue dotted frame). The three primary synthons assemble the one-dimension LSAM (LSAM (1D)), as shown in **Figure S4c)**. The LSAMs (1D) assemble into corrugated close-packing two-dimension LSAM (LSAM (2D)), as shown in **Figure S4d)**. And two corrugated PP molecules chains form two herringbone-type fashion in opposite direction. The three patterns of synthons are arranged at 90° to each other, forming a cyclic tetramer comprising of one PC and three PP molecules. The 1D/2D LSAMs in **PC_PP** cocrystal are quite different from 1D/2D LSAMs in **MC_PP** cocrystal and **OC_PP** cocrystal. The types of supramolecular synthons and intermolecular interaction are also summarized in **Table 1**.

S3.1.2. Hirshfeld surface (HS) analysis and intermolecular interaction modes

Hirshfeld surface (HS) analysis is a useful tool for the quantitative analysis and an unbiased identification for fundamental discussion of the intermolecular interactions of all close contacts. Since the Hirshfeld surface is the electron density isosurface defined by the molecule and the proximity of

its nearest neighbours, it can provide direct insight into intermolecular interactions in crystals (Spackman & Jayatilaka, 2009; Spackman *et al.*, 2008; Ravat *et al.*, 2015). The Hirshfeld surface emerged from an attempt to define the space occupied by a molecule in a crystal for the purpose of partitioning the crystal electron density into molecular fragments (Spackman & Byrom, 1997). Generally, molecular Hirshfeld surfaces can be constructed by partitioning space in the crystal into regions where the electron distribution of a sum of spherical atoms for the molecule (the promolecule) dominates the corresponding sum over the crystal (the procrystal) (McKinnon *et al.*, 2004). Using the HS analysis, a comparative analysis of intermolecular interaction in clusters and monomers was performed. The results are shown in **Figure S5**, and **Table 1**.

HS analysis (Hirshfeld surface mapped with d_{norm} – a function that highlights contact distances relative to the sum of van der Waals radii, with closest contacts shown in red – and fingerprint plots) indicates that two significant and obvious secondary interactions such as O-H···N and N-H···π supramolecular heterosynthon contacts are involved in **MC_PP** cocrystal and **OC_PP** cocrystal formed by two MC molecules or two OC molecules and one PP molecule with the bond length of 1.860 Å and 2.458 Å for **MC_PP** cocrystal and 1.887 Å and 2.442 Å for **OC_PP** cocrystal, respectively. In **PC_PP** cocrystal, except for O-H···N and N-H···π supramolecular heterosynthons contacts formed by one PC molecule and one PP molecule with the bond length of 1.83 Å and 2.431 Å, one more supramolecular homosynthon contacts, *e.g.* N-H···N formed by two PP molecules with the bond length of 2.15 Å was also found. The hydrogen bonding lengths of the supramolecular heterosynthon (O-H···N and N-H···π) in **PC_PP** are much shorter than those in **MC_PP** and **OC_PP** cocrystals. Therefore, it can be inferred that heterotrimers with the types of O-H···N and/or N-H···π potentially exist in the structures prior to the formation of **MC_PP** and **OC_PP** cocrystals while the hetero- or homodimers with the type of O-H···N and/or N-H···π and/or N-H···N are most likely to exist in structures prior to the formation of **PC_PP** cocrystals, as shown in **Figure S4d)**. Moreover, since the hydrogen bonding length d(H···N, O-H···N) < d(H···N, N-H···N) < d(H···π), the O-H···N and N-H···N are moderately strong hydrogen bonding while the N-H···π is weak hydrogen bonding. Therefore, it is highly possible that the π···H hydrogen bonding is formed in order to enhance the stability of solid during the cocrystal formation. And the strength of single hydrogen bonding formed by one cresol and piperazine molecule in **PC_PP** cocrystal might be much stronger than those in **MC_PP** and **OC_PP** cocrystals. Thus, the results obtained from HS analysis are basically consistent with cocrystal structure analysis: the three cresol isomer cocrystals exhibit consistency in the main weak interaction types, that is, they all interact with O-H···N hydrogen bonding and N-H···π hydrogen bonding. However, one more weak interaction of N-H···N hydrogen bonding with strength in between the strength of O-H···N and N-H···π hydrogen bonding was also found in **PC_PP** cocrystal, which is the variability of the **PC_PP** cocrystal at the weak interaction level. In order to

further verify these hypotheses, spectrum and energy analysis of these potential synthons were performed both by experiments and by simulation using Gaussian 09.

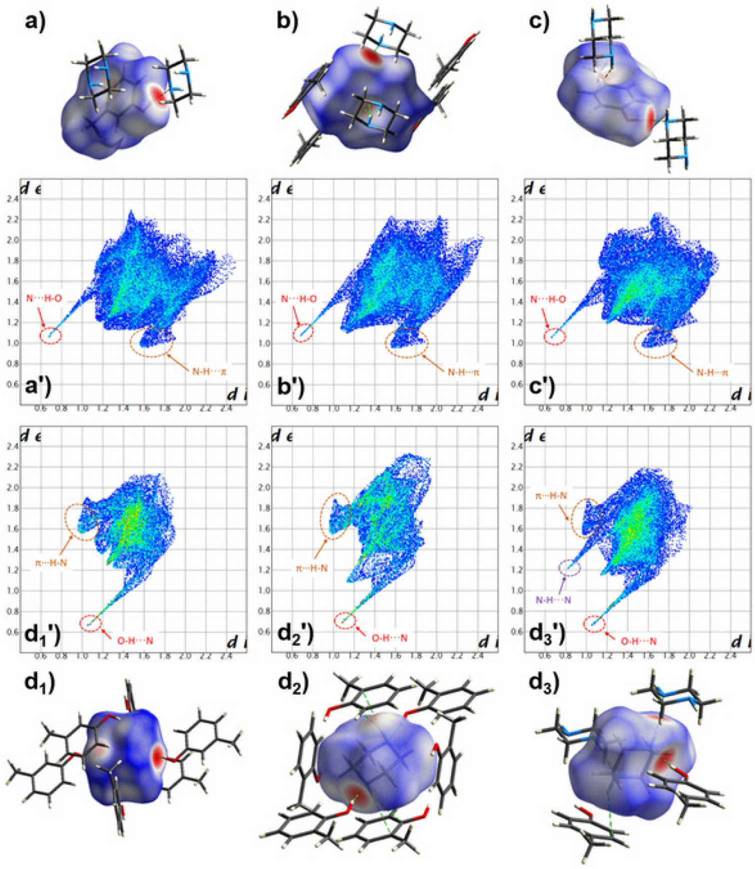

Figure S5 Hirshfeld surface for **MC_PP**, **OC_PP** and **PC_PP** cocrystals mapped with d_{norm} respectively, and neighbouring molecules associated with close contacts are shown between the atoms involved. And the comparison between fingerprint plots for the single molecule in **MC_PP**, **OC_PP** and **PC_PP** cocrystals, respectively. Different features characteristic of key intermolecular contacts is circled in different colours: red circles for the O-H···N hydrogen bonding, orange circles for the N-H···π hydrogen bonding, and purple circle for the N-H···N hydrogen bonding, respectively. (a), a'), d1), d1')) is for **MC_PP** cocrystal, (b), b'), d2), d2')) is for **OC_PP** cocrystal, and (c), c'), d3), d3')) is for **PC_PP** cocrystal, respectively.

S3.2. Possible self-assembly patterns of supramolecular synthons in solution

S3.2.1. IR features of various synthons

Figure S6a-1) shows the solid FTIR spectra of **MC_PP** cocrystal, MC and PP respectively. The black curve represents the experimental result and light purple curve represents the computed **MC_PP(Cal)** synthon with "O(1)-H(1D)···N(1)" hydrogen bonding in gas. The light blue curve represents the computed **PI_MC_PP(Cal)** synthon with "N(1)-H(1)···π" hydrogen bonding in gas. The **MC_PP** cocrystal shows an obvious hydroxyl stretching vibration ($\nu_{O\text{-}H}$) at 3297 cm^{-1}, which is significantly

red-shifted compared with that of MC at 3312 cm^{-1}. This means that the OH groups in MC act as hydrogen bonding donors to form strong hydrogen bonding with strongly electronegative groups, *e.g.* NH of PP. **Figure 3** displays the vibration modes of the infrared fingerprint region. For the black solid curve (ATR-FTIR data, liquid), the heterotrimers (it will be proved later in ^{1}H NMR analysis) shows an obvious C-O stretching vibration ($v_{C\text{-}O}$) at 1285 cm^{-1} in toluene, and the calculation results of two types of heterotrimers, which are **t(MCPP)** combined with O(1)-H(1D)···N(1) and **t(PI_MCPP)** combined with N(1)-H(1)···π, also show that there is strong C-O stretching vibration at 1266 cm^{-1} for **t(MCPP)** and 1267 cm^{-1} for **t(PI_MCPP)**, respectively. Comparing with the light purple line (the computed results of **t(MCPP)** in toluene) and the experimental results, the light grey line (the computed results of **t(PI_MCPP)** in toluene) lacks many obvious and useful characteristic peaks. In particular, there is no O-H in-plane bending vibration ($\delta_{O\text{-}H}$) at around 1482 cm^{-1} and 1353 cm^{-1}. In contrast, the light purple line (computed results of **t(MCPP)** in toluene) shows more and stronger vibration modes than those of **t(PI_MCPP)**, which is in good agreement with the experimental results. What is more, there are O-H in-plane bending vibration ($\delta_{O\text{-}H}$) corresponding to the experimental data at 1473 cm^{-1} and 1351 cm^{-1}, respectively, in the light purple line. For the verification of evolution path of hetero-dimers and/or trimers during cocrystal formation by PAT tool, the ATR-FTIR peak at 1591 cm^{-1} ($v_{C=C,\ Ring}$) was chosen to represent MC, peak at 1319 cm^{-1} ($\delta_{C\text{-}H,\ CH2}$) was chosen to represent liquid PP and peak at 1285 cm^{-1} ($v_{C\text{-}O}$) was chosen to represent **t(MCPP)**, respectively, as shown in **Figure 3**. As to **OC_PP** cocrystal formation, the ATR-FTIR peak at 1174 cm^{-1} ($v_{C\text{-}O}$) was chosen to represent OC, peak at 1137 cm^{-1} ($v_{as(C\text{-}N)}$) was chosen to represent liquid PP and peak at 1252 cm^{-1} ($v_{C\text{-}O}$) was chosen to represent **t(OCPP)**, respectively. And as to PC_PP cocrystal formation, the ATR-FTIR peak at 1602 cm^{-1} ($v_{C=C,\ Ring}$) was chosen to represent PC, peak at 1267 cm^{-1} ($\delta_{C\text{-}H,\ CH2}$) was chosen to represent liquid PP and peak at 1270 cm^{-1} ($\delta_{C\text{-}H,\ CH2}$) was chosen to represent **d(PCPP)**, respectively.

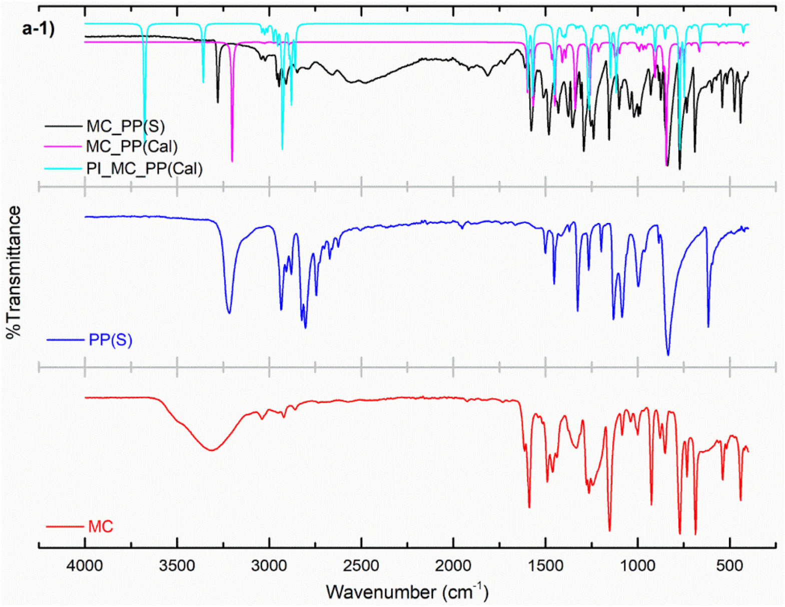
a-1)
MC_PP(S)
MC_PP(Cal)
PI_MC_PP(Cal)
%Transmittance
PP(S)
MC
4000
3500
3000
2500
2000
1500
1000
500
Wavenumber (cm-1)

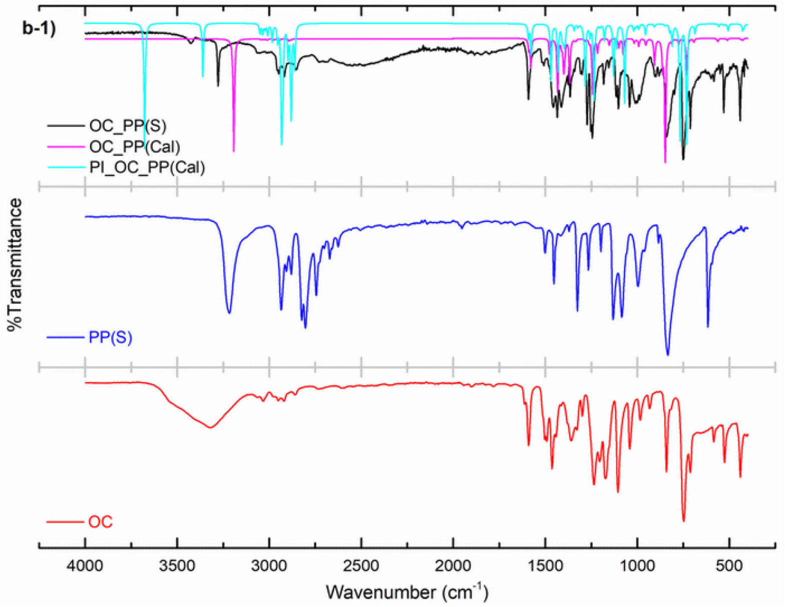
b-1)
OC_PP(S)
OC_PP(Cal)
PI_OC_PP(Cal)
%Transmittance
PP(S)
OC
4000
3500
3000
2500
2000
1500
1000
500
Wavenumber (cm-1)

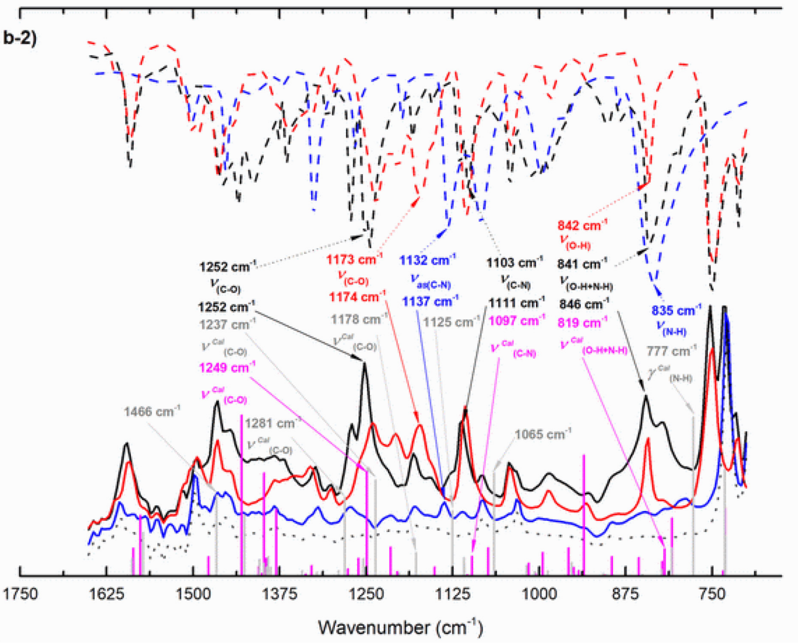
b-2)
1252 cm-1
ν(C-O)
1173 cm-1
ν(C-O)
1132 cm-1
νas(C-N)
1103 cm-1
ν(C-N)
842 cm-1
ν(O-H)
841 cm-1
ν(O-H+N-H)
1252 cm-1
1237 cm-1
νCal(C-O)
1249 cm-1
νCal(C-O)
1174 cm-1
1178 cm-1
νCal(C-O)
1137 cm-1
1125 cm-1
1111 cm-1
1097 cm-1
νCal(C-N)
846 cm-1
819 cm-1
νCal(O-H+N-H)
835 cm-1
ν(N-H)
777 cm-1
γCal(N-H)
1466 cm-1
1281 cm-1
νCal(C-O)
1065 cm-1
1750
1625
1500
1375
1250
1125
1000
875
750
Wavenumber (cm-1)

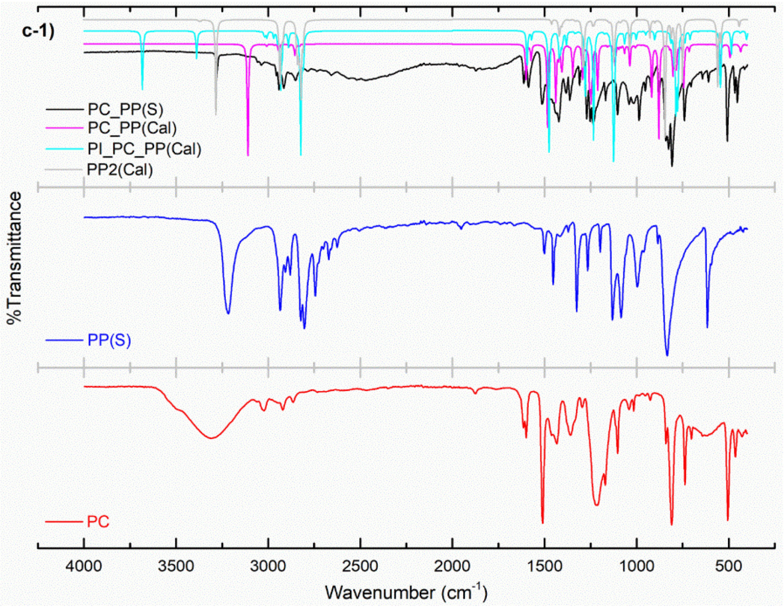
c-1)
PC_PP(S)
PC_PP(Cal)
PI_PC_PP(Cal)
PP2(Cal)
%Transmittance
PP(S)
PC
4000
3500
3000
2500
2000
1500
1000
500
Wavenumber (cm-1)

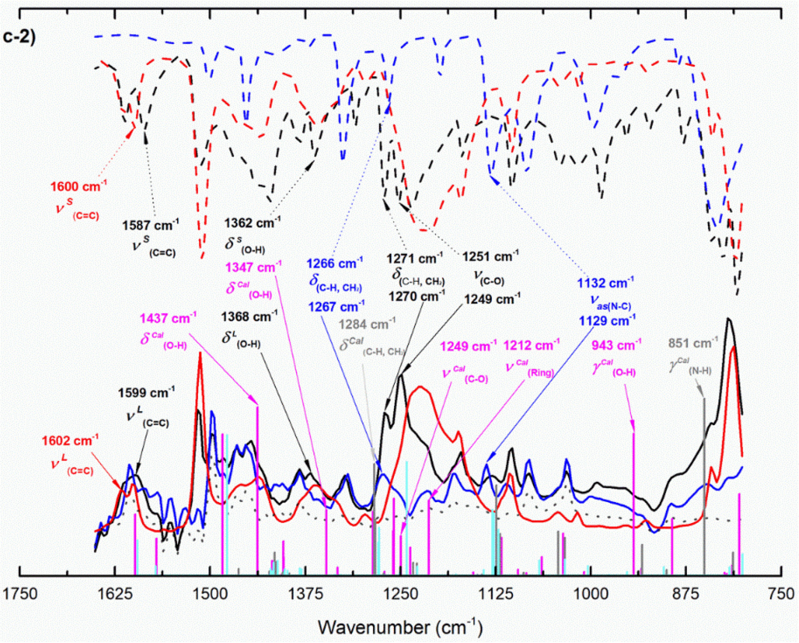

Figure S6 FTIR spectra of **MC_PP**, **OC_PP** and **PC_PP** cocrystals, MC/OC/PC and PP respectively.

a-1) The solid FTIR spectra of **MC_PP** cocrystal (experimental: black, computational: light purple for **MC_PP(Cal)**; light blue for **PI_MC_PP(Cal)**, respectively), MC and PP.

b-1) The solid FTIR spectra of **OC_PP** cocrystal (experimental: black, computational: light purple for **OC_PP(Cal)**; light blue for **PI_OC_PP(Cal)**, respectively), OC and PP. b-2) The characteristic absorption peaks corresponding to solid FTIR data (dotted black curve for **OC_PP** cocrystal, red for OC and blue for PP, respectively), liquid ATR-FTIR data (solid black curve for **OC_PP trimer**, red for OC and blue for PP in toluene, and grey dotted curve for toluene, respectively) and the computational results in toluene (light purple vertical line for the **t(OCPP)** in toluene solution and light grey vertical line for the **t(PI_OCPP)** in toluene solution, respectively)

c-1) The solid FTIR spectra of **PC_PP** cocrystal (experimental: black, computational: light purple for **PC_PP(Cal)**; light blue for **PI_PC_PP(Cal)**; light grey for **PP2**, respectively), PC and PP. c-2) The characteristic absorption peaks corresponding to solid FTIR data (dotted black curve for **PC_PP** cocrystal, red for PC and blue for PP, respectively), liquid ATR-FTIR data (solid black curve for **PC_PP** dimer, red for PC and blue for PP in toluene, and grey dotted curve for toluene, respectively) and the computational results in toluene (light purple vertical line for the **d(PCPP)** in toluene solution, light grey vertical line for the **d(PP2)** in toluene solution, and the light blue vertical line for the **d(PI_PCPP)** in toluene solution, respectively)

(Symbols: v: Stretching, δ: In-Plane Bending; Superscripts: ^{L}: ATR-FTIR data, ^{S}: FTIR data, ^{cal}: computational data; Subscripts: $_{as}$: Antisymmetric)

S3.2.2. Raman features

Figure S7 shows the Raman spectra of **MC_PP**, **OC_PP** and **PC_PP** cocrystal (black solid lines), MC/OC/PC (red solid lines) and PP (blue solid lines), respectively. It can be seen from **Figure S7** that the Raman peak intensity of OH of MC/OC/PC group and HN group of PP are not particularly strong and the peak positions of them are partially overlapped. Therefore, in this work, for the **MC_PP** cocrystal formation, the Raman peak at 1442 cm^{-1} ($\delta_{O\text{-}H}$) was chosen to represent **MC_PP** cocrystal while Raman peaks at 1584 cm^{-1} ($\nu_{C=C}$) and 827 cm^{-1} (ν_{Ring}) were chosen to represent MC and PP, respectively. For the **OC_PP** cocrystal formation, the Raman peak at 1274 cm^{-1} ($\delta_{C\text{-}H,\ CH2}$) was chosen to represent **OC_PP** cocrystal while Raman peaks at 1442 cm^{-1} ($\delta_{O\text{-}H}$) and 1299 cm^{-1} ($\delta_{C\text{-}H,\ CH2}$) were chosen to represent OC and PP, respectively. And for the **PC_PP** cocrystal formation, the Raman peak at 1273 cm^{-1} ($\delta_{C\text{-}H,\ CH2}$) was chosen to represent **PC_PP** cocrystal while Raman peaks at 1600 cm^{-1} ($\nu_{C=C}$) and 1299 cm^{-1} ($\delta_{C\text{-}H,\ CH2}$) were chosen to represent PC and PP, respectively. The characteristic peaks tracked during the PAT process are noted on the line.

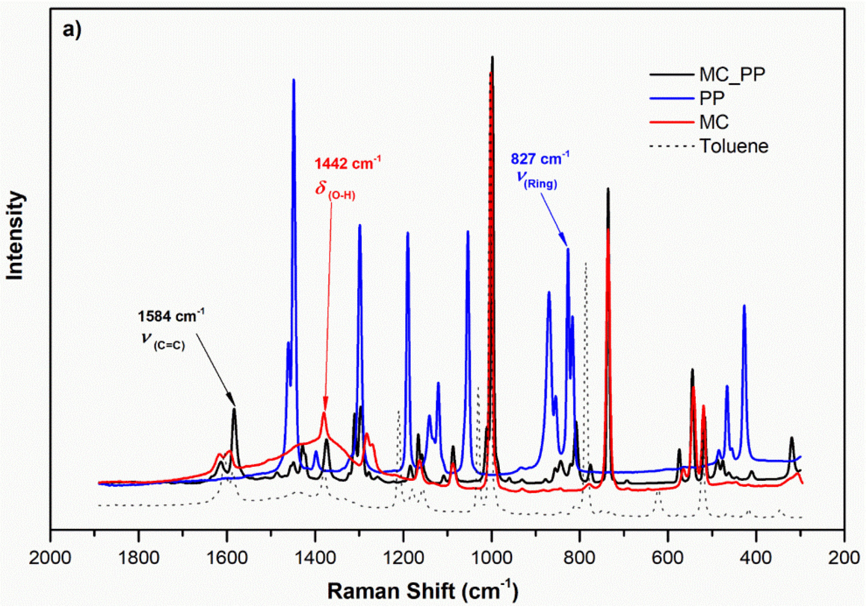

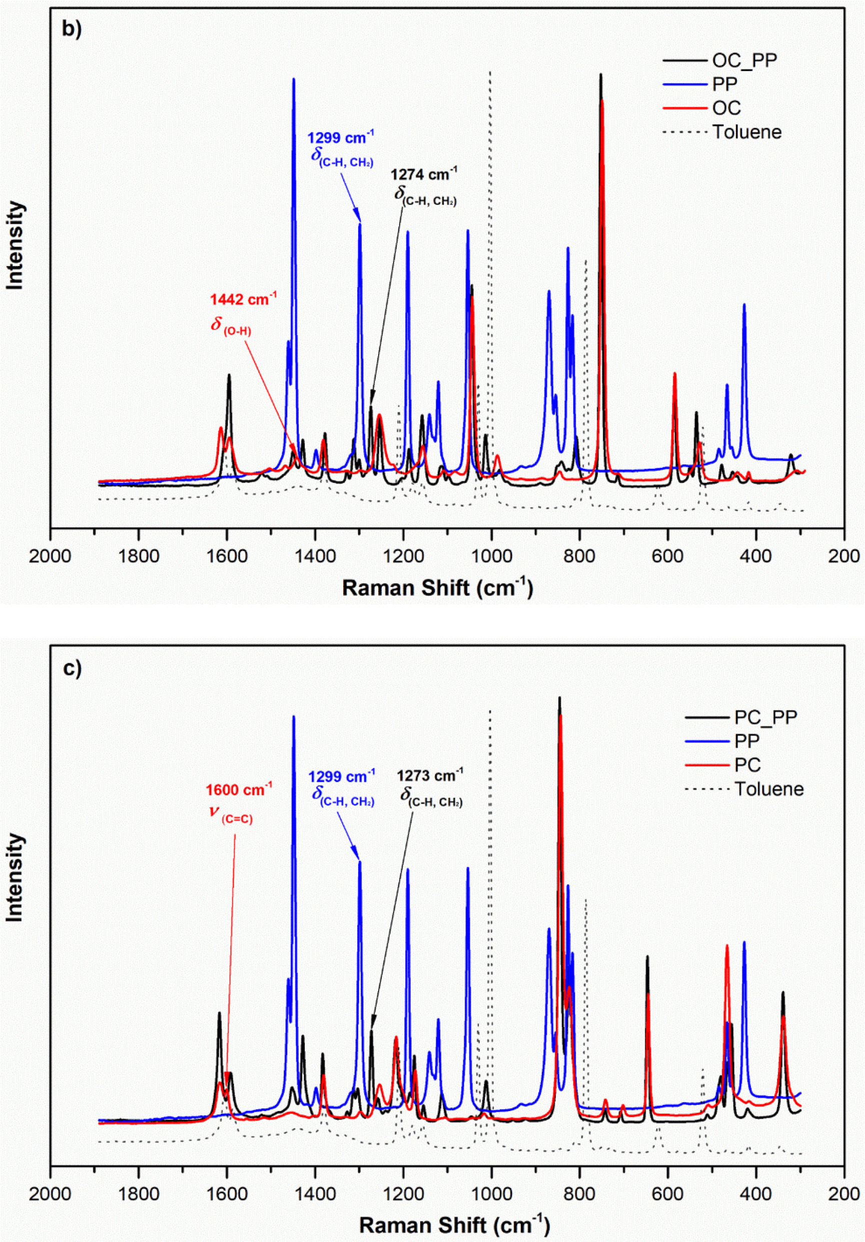

Figure S7 Raman spectra of **MC_PP**, **OC_PP** and **PC_PP** cocrystal, MC/OC/PC and PP respectively. The characteristic peaks tracked during the PAT process are noted on the line.

(Symbols: ν: Stretching, δ: In-Plane Bending; Subscripts: Ring: Benzene ring of toluene)

S3.2.3. ^{1}H NMR features of various synthons

The ^{1}H NMR spectra of **MC_PP** cocrystal, MC and PP is taken as an example to illustrate the results and the assignments of protons signals are shown in **Figure 4**. Since intermolecular hydrogen bonding (O-H···N) is formed between the MC and PP molecules in toluene, the hydrogen of hydroxyl on MC (**MC_PP** cocrystal) is affected by covalent bond and hydrogen bonding, which are both electronegative groups acting as the double inductive effect of electron-withdrawing for the hydrogen. This effect makes the protons involved in hydrogen bonding more exposed than those which do not

form hydrogen bonding and eventually leads to a stronger deshielding effect and lower resonance field, meaning that the chemical shift of protons is increased. From **Figure 4**, it can be seen that the chemical shift of OH on MC increases from 4.10 ppm to 4.90 ppm due to the formation of hydrogen bonding between N-H (PP) and O-H (MC) in the cocrystal and/or in the heterosynthons in toluene. Additionally, because of the very strong intermolecular O-H···N-H hydrogen bonding formed in toluene, the proton on hydroxyl group of MC is almost completely attracted by the N on the imino-group of the PP (consistent conclusions can also be drawn from the calculated bond length shown in **Table 1**), which leads to great electronegativity of O atom on the hydroxyl group, like a negatively charged group. It also weakens the shielding effect of all protons on MC molecule more or less, leading to the chemical shifts move to low field and increase the chemical shifts. Moreover, the chemical shift of the proton on the imino-group of PP molecule moves to the low field, from 0.91 ppm to 4.90 ppm, due to the formation of O-H···N hydrogen bonding. The reason is that the formation of hydrogen bonding reduces the constraint effect of N on H, and the hydroxy oxygen with strong electronegativity is very easy to interact with the proton on the imino-group thanks to the isotropy of molecules in solution. However, the chemical shifts of the protons in methylene of PP molecule move to the high filed, from 2.54 ppm to 2.31 ppm, due to the enhanced shielding effect caused by the formation of O-H···N hydrogen bonding. For **OC_PP** cocrystal and **PC_PP** cocrystal in toluene solution, the chemical shifts of cresol molecules and PP molecules in their own cocrystals are similar to those of **MC_PP** cocrystal, as shown in **Figure S8** and **Table S3**. Meanwhile, the area of characteristic peaks was normalized to obtain the stoichiometric ratio of the heterosynthons in toluene solution. The results are shown in **Table S3**.

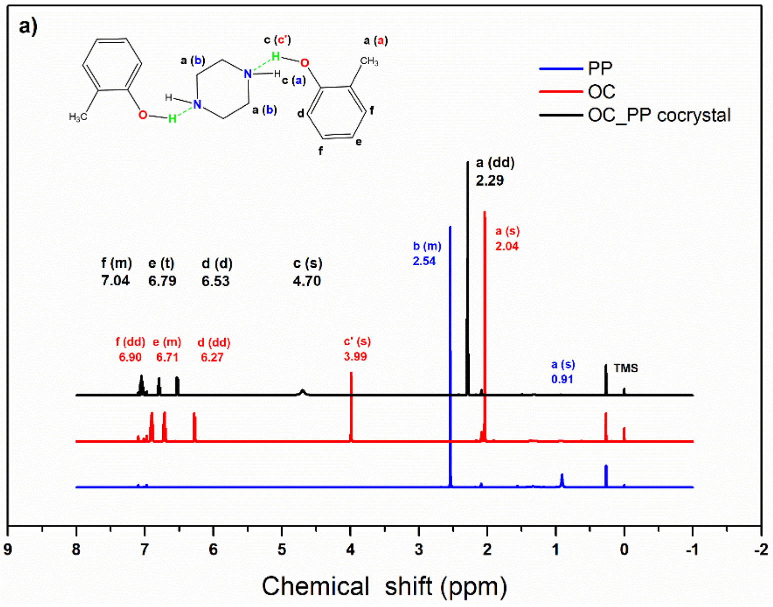

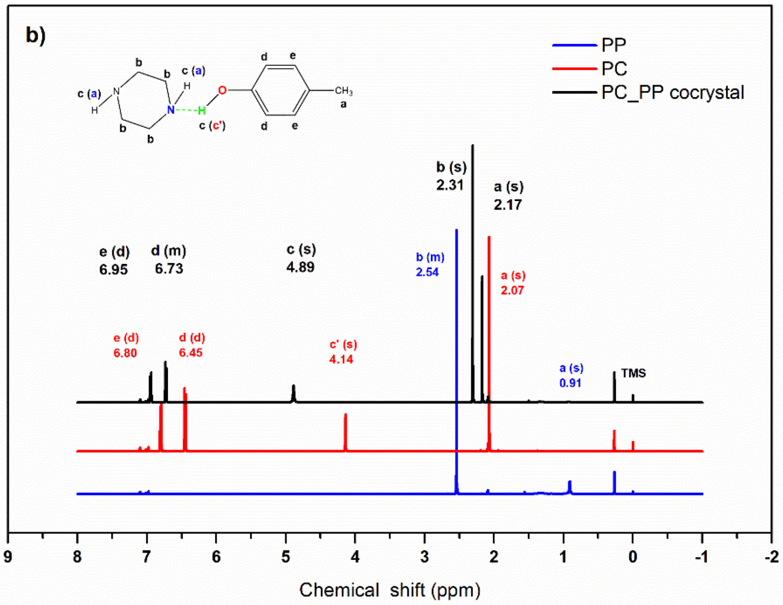

Figure S8 a) ^{1}H NMR spectra of **OC_PP** cocrystal, OC and PP in Toluene-d8, respectively.

^{1}H NMR (OC_PP cocrystal, 500 MHz, [D8]Toluene, 25 °C, TMS): δ = 7.04 (m, 4H; =CH-), 6.79 (t, 2H; =CH-), 6.53 (d, 2H; =CH-),4.70 (s, 4H; OH+NH), 2.29 (s, 14H; CH_2+CH_3).

^{1}H NMR (OC, 500 MHz, [D8]Toluene, 25 °C, TMS): δ = 6.90 (dd, 1H; =CH-), 6.71 (m, 1H; =CH-), 6.27 (dd, 1H; =CH-), 3.99 (s, 1H; OH), 2.04 ppm (s, 3H; CH_3).

^{1}H NMR (PP, 500 MHz, [D8]Toluene, 25 °C, TMS): δ = 2.54 (m, 4H; =CH_2-), 0.91 (s, 1H; NH).

b) ^{1}H NMR spectra of **PC_PP** cocrystal, PC and PP in Toluene-d8, respectively.

^{1}H NMR (PC_PP cocrystal, 500 MHz, [D8]Toluene, 25 °C, TMS): δ = 6.95 (d, 2H; =CH-), 6.73 (m, 2H; =CH-), 4.89 (s, 3H; OH+NH), 2.31 (s, 8H; CH_2), 2.17 ppm (s, 3H; CH_3).

^{1}H NMR (PC, 500 MHz, [D8]Toluene, 25 °C, TMS): δ = 6.80 (d, 2H; =CH-), 6.45 (d, 2H; =CH-), 4.14 (s, 1H; OH), 2.07 ppm (s, 3H; CH_3).

^{1}H NMR (PP, 500 MHz, [D8]Toluene, 25 °C, TMS): δ = 2.54 (m, 4H; =CH_2-), 0.91 (s, 1H; NH).

Table S3 ^{1}H NMR data of cocrystals and their compositions

Components		Assignment	Chemical Shift/ppm	Range ^{a} /ppm	Normalized ^{b}	Normalized 2 ^{c}
1	MC_PP cocrystal	t, 2H; =CH-	7.07	7.11 ~ 7.04	12.95	12.95
		ddd, 6H; =CH-	6.63	6.68 ~ 6.58	34.29	34.29
		s, 4H; OH+NH	4.90	5.00 ~ 4.81	24.69	24.69
		s, 8H; CH_2	2.31	2.33 ~ 2.29	53.28	53.28
		s, 6H; CH_3	2.18	2.20 ~ 2.16	37.24	37.24

	TMS	-	0.00	0.02 ~ -0.02	**1.00** [d]	-
2	OC_PP cocrystal	m, 4H; =CH-	7.04	7.09 ~ 6.98	27.16	27.16
		t, 2H; =CH-	6.79	6.83 ~ 6.76	12.74	12.74
		d, 2H; =CH-	6.53	6.56 ~ 6.50	13.19	13.19
		s, 4H; OH+NH	4.70	5.06 ~ 4.34	23.75	23.75
		dd, 14H; CH_2+CH_3	2.29	2.31 ~ 2.26	103.08	103.08
	TMS	-	0.00	0.02 ~ -0.02	**1.00** [d]	-
3	PC_PP cocrystal	d, 2H; =CH-	6.95	6.97 ~ 6.92	28.51	28.51
		m, 2H; =CH-	6.73	6.75 ~ 6.70	28.32	28.32
		s, 3H; OH+NH	4.89	4.98 ~ 4.79	30.17	30.17
		s, 8H; CH_2	2.31	2.33 ~ 2.29	64.47	64.47
		s, 3H; CH_3	2.17	2.19 ~ 2.15	45.95	45.95
	TMS	-	0.00	0.02 ~ -0.02	**1.00** [d]	-
4	MC	dd, 1H; =CH-	6.94	7.00 ~ 6.90	13.81	10.72
		d, 1H; =CH-	6.56	6.59 ~ 6.53	12.19	9.46
		dd, 1H; =CH-	6.35	6.39 ~ 6.32	12.12	9.41
		s, 1H; =CH-	6.30	6.32 ~ 6.27	12.06	9.36
		s, 1H; OH	4.10	4.12 ~ 4.08	11.90	9.24
		s, 3H; CH_3	2.06	2.07 ~ 2.04	40.24	31.24
	TMS	-	0.00	0.02 ~ -0.02	**1.00** [d]	-
5	OC	dd, 2H; =CH-	6.90	6.95 ~ 6.86	21.42	19.97
		m, 1H; =CH-	6.71	6.75 ~ 6.68	10.73	10.00
		dd, 1H; =CH-	6.27	6.03 ~ 6.25	10.42	9.72
		s, 1H; OH	3.99	4.01 ~ 3.96	10.24	9.55
		s, 3H; CH_3	2.04	2.06 ~ 1.99	34.76	32.41
	TMS	-	0.00	0.02 ~ -0.02	**1.00** [d]	-
6	PC	d, 2H; =CH-	6.80	6.83 ~ 6.77	27.30	20.85
		d, 2H; =CH-	6.45	6.48 ~ 6.41	27.03	20.64
		s, 1H; OH	4.14	4.17 ~ 4.11	12.86	9.82
		s, 3H; CH_3	2.07	2.08 ~ 2.06	47.68	36.41
	TMS	-	0.00	0.02 ~ -0.02	**1.00** [d]	-
7 MC_PP	PP	m, 8H; CH_2	2.54	2.56 ~ 2.51	199.19	50.33
		s, 2H; NH	0.91	0.95 ~ 0.88	57.24	14.46
	TMS	-	0.00	0.02 ~ -0.02	**1.00** [d]	-
7 OC_PP	PP	m, 8H; CH_2	2.54	2.56 ~ 2.51	199.19	53.69
		s, 2H; NH	0.91	0.95 ~ 0.88	57.24	15.43

	TMS	-	0.00	0.02 ~ -0.02	**1.00** [d]	-
7 PC_PP	PP	m, 8H; CH_2	2.54	2.56 ~ 2.51	199.19	78.82
		s, 2H; NH	0.91	0.95 ~ 0.88	57.24	22.65
	TMS	-	0.00	0.02 ~ -0.02	**1.00** [d]	-

[a]: Integral range of a certain characteristic peak.

[b]: Normalized values compared with the standard peak is processed by MestReNova software. In this process, the peak of TMS was selected as the standard peak.

[c]: Normalized values at the same concentration compared with the standard peak. (Wang *et al.*, 2017)

[d]: Standard peak, and the normalized value of the standard peak is 1.00.

S3.2.4. Intermolecular interaction energy of synthons.

From **Table 2**, it can be seen that the interaction energies of all types of supramolecular synthons in the toluene solvent are higher than those of the corresponding synthons in gas-phase, which indicates that the solvation layers formed around a single molecule hinder the formation of hydrogen bonding and are not conducive to the formation of hydrogen bonding between two molecules. When the solvated molecules interact with each other to form dimers or trimers, the repulsive interaction of the solvation layers must be overcome first. In addition, the interaction energies of the heterodimers or heterotrimers combined with the π···H (N-H···π) hydrogen bonding are the highest, even higher than those of homodimers with the N-H···N hydrogen bonding, indicating that the dominant synthon are not the heterosynthons with the π···H (N-H···π) hydrogen bonding. Moreover, the energy of synthons with π···H (N-H···π) hydrogen bonding in the toluene solvent is significantly higher than that of synthons with π···H (N-H···π) hydrogen bonding in gas-phase while the difference of the O-H···N hydrogen bonding energy in toluene solvent and in gas-phase is far less than the difference of N-H···π hydrogen bonding energy. It indicates that the O-H···N hydrogen bonding is preferentially formed in the solvent and the N-H···π (and N-H···N for **PC_PP** cocrystal) hydrogen bonding is probably further formed in the process of cocrystal formation. Additionally, the energy of supramolecular synthons between **MC_PP** cocrystal and **OC_PP** cocrystal is very close. From the interaction energy point of view, the formation difficulty and stability of **MC_PP** and **OC_PP** are consistent. The lattice energy of **MC_PP** cocrystal and **OC_PP** cocrystal is almost the same, which can explain why the melting point of **MC_PP** cocrystal and **OC_PP** cocrystal is very close, as shown in **Figure S11**. Nevertheless, the energy of the supramolecular synthons of **PC_PP** cocrystal is obviously different from the corresponding supramolecular synthons of **MC_PP** and **OC_PP** cocrystal, which shows variability in energy perspective. Compared with **MC_PP** cocrystal and **OC_PP** cocrystal, the energy of binary heterosynthons of **PC_PP** cocrystal is lower than the half of interaction energy of ternary heterosynthons of **MC_PP** cocrystal and **OC_PP** cocrystal. In other word, for the $\Delta \boldsymbol{E}_{\mathbf{d}}$ column, the supramolecular synthons interaction energy of the **PC_PP** cocrystal are the lowest in both gas-phase

and toluene solution, ΔE_d: **I**(PC_PP) < **I**(OC_PP) ≈ **I**(MC_PP) < **III**(PC_PP) < **II**(PC_PP) < **II**(MC_PP) ≈ **II**(OC_PP). This is most likely related to the number of interacting cresol molecules with PP molecules and the reasons would be explained later. Meanwhile, the lattice energy of **PC_PP** cocrystal is also the largest, resulting in the melting point of it higher than the **MC_PP** cocrystal and **OC_PP** cocrystal, as shown in **Figure S11**, and it can also be inferred that the PC and PP molecules in **PC_PP** cocrystal are more closely packed.

S3.2.5. Verification of evolution path of hetero-dimers and/or trimers during cocrystal formation by PAT tool

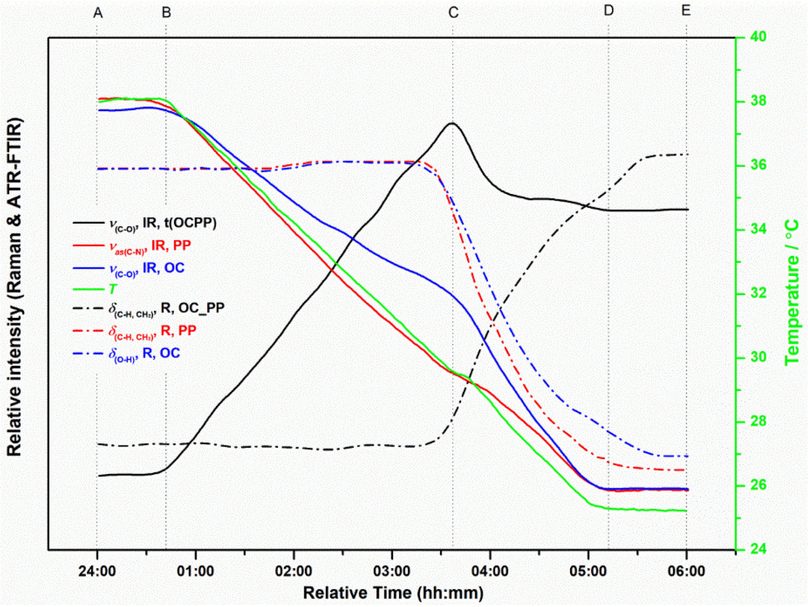

Figure S9 Changing trends of Raman and ATR-FTIR data during cooling crystallization process of trimer verification experiments for **OC_PP** cocrystal.

(R: Raman data, IR: ATR-FTIR data)

As can be seen from the PAT profiles, when the clarified solutions were cooled down to a certain temperature, the supersaturation of the heterodimers or heterotrimers were accumulated enough to nucleate (from B to C). Hence, the relatively ATR-FTIR intensity of heterodimers or heterotrimers (black solid lines) increased, while those of MC/OC/PC and PP (blue and red solid lines) decreased, as shown in **Figure 9**, **S9** and **S10**. Then, the cocrystals begins to nucleate and growth. Meanwhile, the concentration of the heterotrimers (**t(MCPP)** or **t(OCPP)**) or the heterodimers (**d(PCPP)**) began to decrease (from C to D). The concentration of the heterodimers or heterotrimers didn't change anymore when the final temperature was reached. And the content of the cocrystals was also balanced (from D to E). Therefore, it can be suggested that the heterotrimers or heterodimers were firstly formed in the solution before the nucleation and growth of cocrystals. And the accumulation of

t(MCPP) or **t(OCPP)** or **d(PCPP)** supersaturation was mainly induced by the decreasing of temperature because high temperature is harmful to formation of hydrogen bonding. And when the concentration of the heterotrimers or heterodimers reached high enough to overcome the nucleation energy barrier, the formation of cocrystal began.

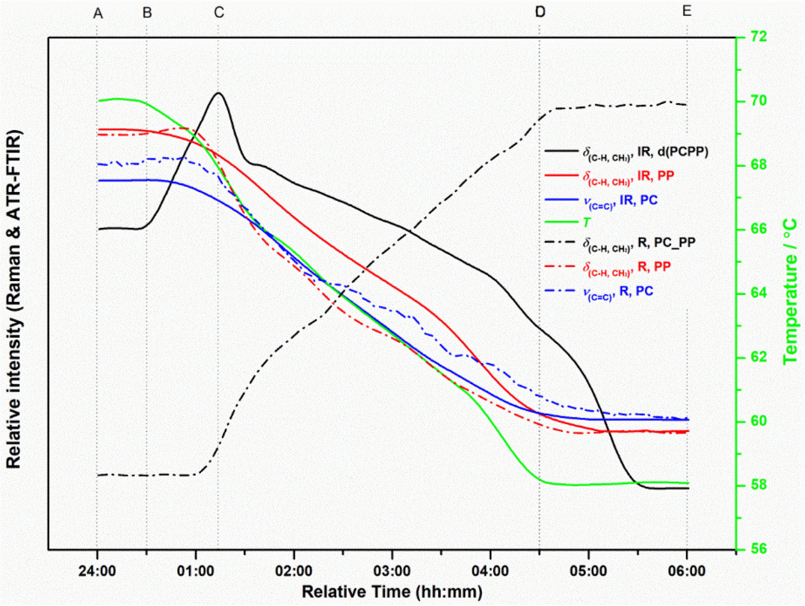

Figure S10 Changing trends of Raman and ATR-FTIR data during cooling crystallization process of trimer verification experiments for **PC_PP** cocrystal.

(R: Raman data, IR: ATR-FTIR data)

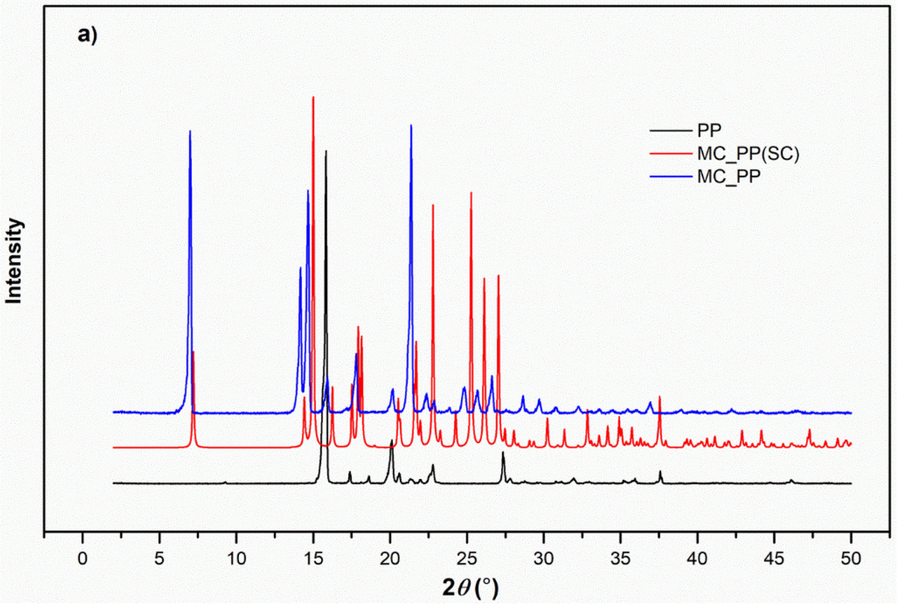

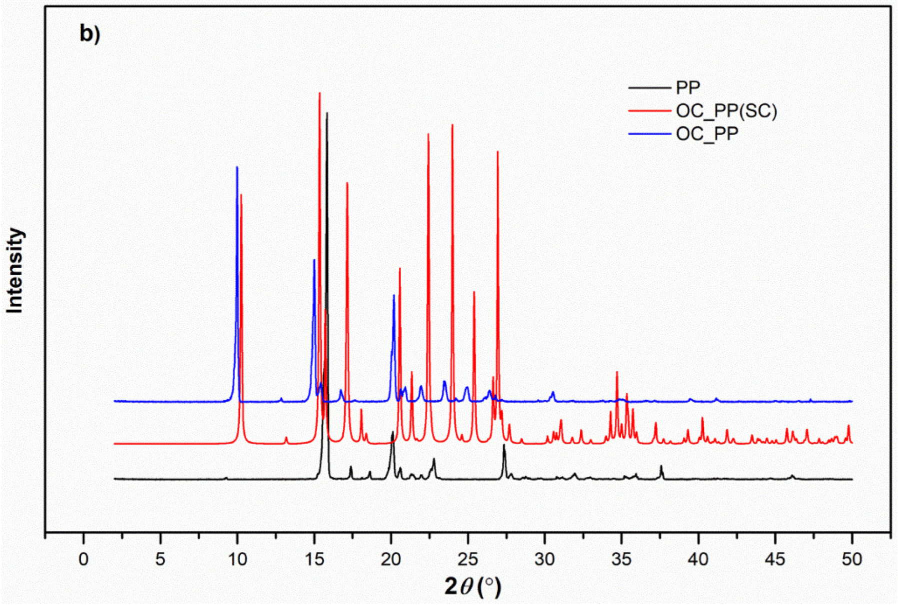
b)
PP
OC_PP(SC)
OC_PP
Intensity
0
5
10
15
20
25
30
35
40
45
50
2θ(°)

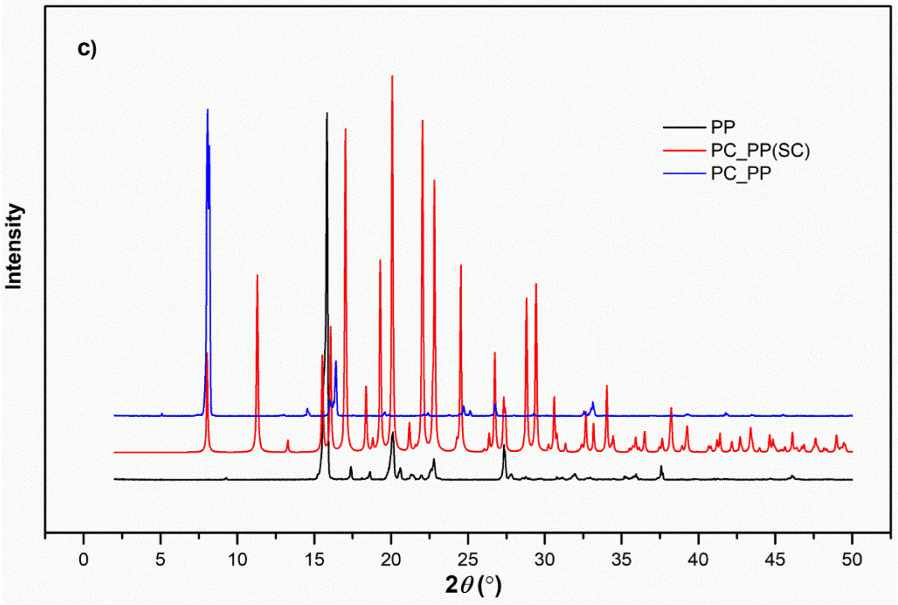
c)
PP
PC_PP(SC)
PC_PP
Intensity
0
5
10
15
20
25
30
35
40
45
50
2θ(°)

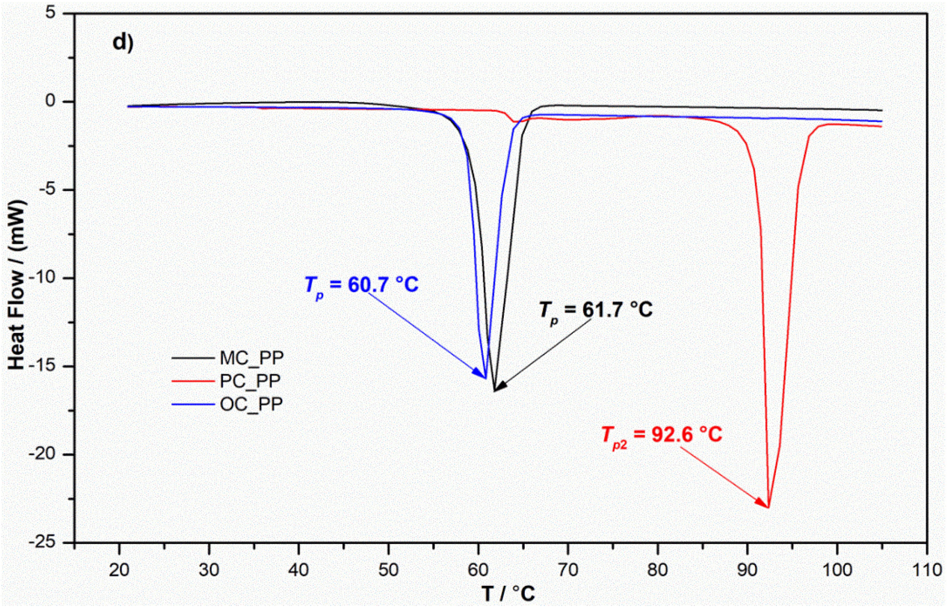

Figure S11 PXRD patterns of a) **MC_PP**, b) **OC_PP** and c) **PC_PP** cocrystals, and DSC curves.

S3.3. CCDC numbers

1906693; 1907006; 1907017

S4. Computational details

All the optimized geometries, the energy evaluation, and vibrational spectra of possible supramolecular synthons' modes of (*m*-, *o*-, *p*-) cresol-piperazine and their monomers were investigated using the DFT-D3 method (Zhao & Truhlar, 2008; Grimme *et al.*, 2010; Lu & Chen, 2013) using the Gaussian09 program package (Frisch *et al.*, 2009), at the M06-2X/6-311+G(d,p) level of theory (Zhao & Truhlar, 2007; Zhao & Truhlar, 2008; Zhao & Truhlar, 2011; Zhao & Truhlar, 2008; Antony *et al.*, 2015). And the “tight” level convergence limits and superfine grid were used in all computations. The initial structures used for geometric optimization are taken from the refined single crystal structure. The cocrystals interaction energies were calculated with the same method, and corrected for the basis set superposition error (BSSE) *via* the standard counterpoise (CP) method of Boys and Bernardi (Zhao & Truhlar, 2007; Boys & Bernardi, 2002). The geometry optimizations, frequency and interaction energies calculations were performed both in the gas-phase as well as in toluene using the SMD (universal solvent model based on the solute electron density) implicit solvation models of Truhlar (Marenich *et al.*, 2009; Tomasi *et al.*, 2005). Additionally, due to the neglecting of anharmonicity effects present in a real system during the frequency calculations and the environment (gas, liquid or solid phase), the calculated frequencies usually differ appreciably from observed frequencies. Therefore, the calculated wavenumbers in the context are scaled down using the

scaling factor 0.9440 (Bao *et al.*, https://comp.chem.umn.edu/ freqscale/version3b2.htm.) for M06-2X/6-311+G(d,p) (Alecu *et al.*, 2010) to disregard the anharmonicity present in the real system.

The lattice energy values (Li *et al.*, 2018; Bisker-Leib & Doherty, 2001) of the cocrystals were performed with Forcite module of Materials Studio (MS) 7.0. The *pcff* force field was selected to optimize the crystal structure and compute the lattice energy. The optimization results of the crystal structure with the *pcff* force field are in good agreement with experimental results (the error of the computation is within 5%), which indicates that the implementation of MD simulations on cocrystals by the *pcff* force field is applicable (Li *et al.*, 2018).

Visualization and confirmation of calculated data were done by using the program GaussView. The ADCH charges (atomic dipole moment corrected Hirshfeld charge) and charge transfers (Lu & Chen, 2012; Lu & Chen, 2012) were calculated by the wavefunction analysis program Multiwfn 3.6v (Lu & Chen, 2012). All isosurface maps were rendered by VMD 1.9.3 program (Humphrey *et al.*, 1996) based on the outputs of Multiwfn 3.6v. And the Hirshfeld surface analysis were available in CrystalExplorer (Turner *et al.*, 2017).

References

a. Spackman, M. A. & Jayatilaka, D. (2009). *CrystEngComm* **11**, 19-32.

b. Spackman, M. A., McKinnon, J. J. & Jayatilaka, D. (2008). *CrystEngComm* **10**, 377–388.

c. Ravat, P., SeethaLekshmi, S., Biswas, S. N., Nandy, P. & Varughese, S. (2015). *Cryst. Growth Des.* **15**, 2389-2401.

d. Spackman, M. A. & Byrom, P. G. (1997). *Chem. Phys. Lett.* **267**, 215-220.

e. McKinnon, J. J, Spackmana, M. A. & Mitchell, A. S. (2004). Acta Cryst. B**60**, 627-668.

f. Wang, N., Hao, H., Lu, H. & Xu, R. (2017). *CrystEngComm.* **19**, 3746-3752.

g. Zhao, Y. & Truhlar, D. G. (2008). *Acc. Chem. Res.* **41**, 157-167.

h. Grimme, S., Antony, J., Ehrlich, S. & Krieg, H. (2010). *J. Chem. Phys.* **132**, 154104-1-154104-19.

i. Lu, T. & Chen, F. (2013). *J. Mol. Model.* **19**, 5387-5395.

j. Frisch, M. J., *et al*. Gaussian 09, Gaussian, Inc., Wallingford CT, 2009. Gaussian.

k. Zhao, Y. & Truhlar, D. G. (2007). *J. Am. Chem. Soc.* **129**, 8440-8442.

l. Zhao, Y. & Truhlar, D. G. (2008). *Theor. Chem. Acc.* **120**, 215-241.

m. Zhao, Y. & Truhlar, D. G. (2011). *Chem. Phys. Lett.* **502**, 1-13.

n. Zhao, Y. & Truhlar, D. G. (2008). *J. Chem. Theory Comput.* **4**, 1849-1868.

o. Antony, J., Sure, R. & Grimme, S. (2015). *Chem. Commun.* **51**, 1764-1774.

p. Boys, S. F. & Bernardi, F. (2002). *Mol. Phys.* **100**, 65-73.

q. Marenich, A. V., Cramer, C. J. & Truhlar, D. G. (2009). *J. Phys. Chem. B.* **113**, 6378-6396.

r. Tomasi, J., Mennucci, B. & Cammi, R. (2005). *Chem. Rev.* **105**, 2999-3093.

s. Bao, J. L., Zheng, J., Alecu, I. M., Lynch, B. J., Zhao Y. & Truhlar, D. G. https://comp.chem.umn.edu/ freqscale/version3b2.htm.

t. Alecu, I. M., Zheng, J., Zhao, Y. & Truhlar, D. G. (2010). *J. Chem. Theory Comput.* **6**, 2872-2887.

u. Li, J., Jin, S., Lan, G., Ma, X., Ruan, J., Zhang, B., Chen, S. & Li, L. (2018). *CrystEngComm* **20**, 6252-6260.

v. Bisker-Leib, V. & Doherty, M. F. (2001). *Cryst. Growth Des.* **1**, 455-461.

w. Lu, T. & Chen, F. (2012). *J. Theor. Comput. Chem.* **11**, 163-183.

x. Lu, T. & Chen, F. (2012). *Acta Phys. -Chim. Sin.* **28**, 1-18.

y. Lu, T. & Chen, F. (2012). *J. Comput. Chem.* **33**, 580-592.

z. Humphrey, W., Dalke, A. & Schulten, (1996). *J. Mol. Graphics* **14**, 33–38.

aa. Turner, M. J., McKinnon, J. J., Wolff,S. K., Grimwood, D. J., Spackman, P. R., Jayatilaka, D. & Spackman, M. A. CrystalExplorer17 (2017), University of Western Australia.
